# New Perspectives on Microbial Community Distortion after Whole-Genome Amplification

**DOI:** 10.1371/journal.pone.0124158

**Published:** 2015-05-26

**Authors:** Alexander J. Probst, Thomas Weinmaier, Todd Z. DeSantis, Jorge W. Santo Domingo, Nicholas Ashbolt

**Affiliations:** 1 Department for Bioinformatics, Second Genome Inc., South San Francisco, California, United States of America; 2 U.S. Environmental Protection Agency, Cincinnati, Ohio, United States of America; Missouri University of Science and Technology, UNITED STATES

## Abstract

Whole-genome amplification (WGA) has become an important tool to explore the genomic information of microorganisms in an environmental sample with limited biomass, however potential selective biases during the amplification processes are poorly understood. Here, we describe the effects of WGA on 31 different microbial communities from five biotopes that also included low-biomass samples from drinking water and groundwater. Our findings provide evidence that microbiome segregation by biotope was possible despite WGA treatment. Nevertheless, samples from different biotopes revealed different levels of distortion, with genomic GC content significantly correlated with WGA perturbation. Certain phylogenetic clades revealed a homogenous trend across various sample types, for instance Alpha- and Betaproteobacteria showed a decrease in their abundance after WGA treatment. On the other hand, *Enterobacteriaceae*, an important biomarker group for fecal contamination in groundwater and drinking water, were strongly affected by WGA treatment without a predictable pattern. These novel results describe the impact of WGA on low-biomass samples and may highlight issues to be aware of when designing future metagenomic studies that necessitate preceding WGA treatment.

## Introduction

Various genomic approaches are now considered fundamental to aid in describing microbial diversity, given that only approximately one out of one thousand microbial species may be cultivated and studied under laboratory conditions [1,2]. Foremost, environmental genomics including metagenomics and single cell sequencing have drastically improved the understanding of the uncultivated majority and its function in ecosystems [3,4,5,6]. Nevertheless, many low-biomass biotopes and their associated microbial lineages and functional potential remain unexplored because of low or undetectable amounts of environmental genomic DNA extracts, whereas approximately 100 nanograms of nucleic acids (of high quality) are necessary to reliably perform next-generation shotgun sequencing.

Low-biomass environments that are of particular interest to the scientific community encompass a wide range of ecosystems and are coupled to various research interests. Subsurface microbial life for instance, was found to be diverse and concomitantly little explored concerning phylogeny and metabolic potential [3,7,8,9,10]. Particularly, groundwater sediments appeared to harbor an extraordinary diversity of many unexplored microbial phyla, which are significantly contributing to carbon and sulfur cycling in these ecosystems [3,9,10]. Clean room facilities represent another low-biomass environment, whose integrity concerning cleanliness is of high interest for pharmaceutical industry and for space agencies. The microbiome composition of multiple clean rooms during spacecraft assembly has been described using 16S rRNA gene amplicon analysis [11,12,13,14,15], yet a metagenomic survey is still to come due to the lack of biomass that can be sampled from such facilities [13]. At the same time, human skin samples are usually low in recovered DNA and even nested-PCR steps are necessary to spot certain microbial taxa that are constant cohabitants on our skin [16]. In general, the functional characterization of low-biomass environments is often hampered by the lack of metagenomic DNA available, which is consequently limiting the knowledge on microbiome function in these biotopes.

The advent of multiple displacement amplification (MDA; also known as whole genome amplification, WGA) with randomized primers under isothermal conditions enables low-biomass samples or those with substantial amounts of PCR-inhibitors to be amplified [17,18,19,20]. For instance, Abulencia et al. showed that the microbial composition assessment of certain contaminated sediment samples was only possible after WGA treatment, since the initial amount of metagenomic DNA was insufficient for PCR amplification [19]. Although WGA provides advantages by increasing DNA template for downstream analyses, the effects of this amplification step can be non-uniform across the genomes in the sample matrix, resulting in distortion to relative abundance composition [21].

The effects of WGA on the microbial community structure that can be reconstructed from the amplified DNA have been studied using both mock-communities and environmental samples [19,21,22]. So far, the results from the aforementioned studies varied greatly, from improved diversity detection after WGA treatment [19] to drastic community shifts [22] to changes detectable on operational taxonomic unit level mainly biasing the highly abundant taxa [21]. Earlier studies focused on low-biomass samples and conventional cloning of 16S rRNA genes for investigating the effects of WGA treatment on microbial communities [17,18,19,20]. These studies reported mainly on the benefits of WGA resulting in improved microbiome assessment. In contrast, more recent surveys used pyrotag sequencing of 16S rRNA genes isolated exclusively from high-biomass environments like activated sludge and reported on the strong biases introduced by WGA [21,22]. Consequently, it remains elusive if the nature of the analysis technology or the nature of the sample’s community composition is responsible for the different pictures that have been reported for WGA effects on microbial community structures.

Here we report on samples taken from five different environments, including low- and high-biomass samples: groundwater (12 biological replicates), drinking water (4 biological replicates), biofilms (7 biological replicates), sludge (4 biological replicates) and municipal biosolids (4 biological replicates). DNA of each biological replicate of each environment was either i) not WGA treated or, ii) added to a WGA reaction at 0.4 ng template mass or, iii) added to a WGA reaction at 4.0 ng template mass. These three conditions are hereafter abbreviated as “pre”, “low”, and “high” WGA treatments and were performed for every biological replicate, resulting in a dataset of 93 samples in total (31 biological replicates with three different WGA treatments each). The effects of WGA treatment were analyzed using PhyloChip G3 DNA microarray technology based 16S rRNA gene amplicons, which has proven to be highly reproducible in previous studies [23]. When the same set of gDNA samples were analyzed by next generation sequencing and PhyloChip, the PhyloChip uncovered a greater diversity of significantly perturbed microbial taxa [24], likely due to the limitation of NGS redundantly sequencing the dominant taxa and not quantitatively observing reads from the minority taxa [25]. In this study, we were interested in quantifying potential distortions in both the majority *and* minority populations in these communities. Our analyses shed light onto the ambiguous nature of WGA treatment on microbial community structures and its dependency on the sample type analyzed. These results will have direct implications on future metagenomic studies of low-biomass environments and facilitate the sensitivity of researchers to investigate the effects of sample handling prior to performing exhaustive sequencing efforts.

## Material and Methods

### Sampling material

The sample manifest consisted of five samples taken from five different biotopes: Groundwater (C, 12 biological replicates), drinking water (D, 4 biological replicates), epilithic microbial community (“biofilms”, N, 7 biological replicates), raw sewage sludge (S, 4 biological replicates), and treated biosolids (T, 4 biological replicates). While groundwater and drinking water were of low-biomass nature, other biotopes were considered representative of high biomass samples as determined by the amount of volume filtered and the DNA extraction yields per sample. Sample collection was restricted to epilithic biomass; no specific permissions from state or local authorities were required for these locations/activities as the field studies did not include collection of animals (vertebrates or invertebrates) or involve endangered or protected species.

### Sampling, sample processing, metagenomic DNA measurement

As mentioned above, samples from five different matrices were used in this study. Groundwater samples (C) consisted of water collected from observation wells located in east-central Illinois and processed as described elsewhere [26]. Drinking water samples (D) were obtained by filtering 1 l of water onto 0.2 μm polycarbonate membranes (GE Osmonics, Minnetonka, MN); filtered biomass was then processed as described by Revetta et al. [27]. Epilithic samples (N) were harvested from biofilms established on unglazed clay tiles submerged on an urban creek in Cincinnati, Ohio (USA). Processing details for epilithic biomass samples are described elsewhere [28]. Raw sewage sludge (S) and treated biosolids (T) samples were obtained from a wastewater treatment plant receiving domestic waste. DNA was isolated from sludge and biosolid samples using UltraClean Soil DNA kit following the manufacturer’s instructions (MoBio Laboratories, Solana Beach, CA). DNA yield was measured by using a Qubit fluorometer as per the manufacturer’s instructions (Life Technologies).

### Whole-genome amplification

Whole genome amplification was carried out using the GenomiPhi DNA Amplification Kit (GE Healthcare Life Sciences, Piscataway, US). This kit uses the Phi29 DNA polymerase and random hexamer primers (composition proprietary according to company information) for amplification of template DNA. All samples investigated herein were used for whole genome amplification (WGA) with 0.4 ng template and 4.0 ng template. In sum, a sample set consisted of three specimens of the same sample, two WGA treated samples (low and high) and an untreated control sample (pre).

### Amplicon generation and microarray hybridization

Bacterial 16S rRNA gene amplicons were generated from each sample individually using the degenerate forward primer 27F.1 (5’-AGRGTTTGATCMTGGCTCAG-3’) and the non-degenerate reverse primer 1492R.jgi (5’-GGTTACCTTGTTACGACTT-3’) [29] via 35 PCR cycles. Amplicons were individually concentrated using a solid-phase reversible immobilization method for the purification of PCR products and quantified by electrophoresis using an Agilent 2100 Bioanalyzer. PhyloChip Control Mix was added to each of the 500 ng of amplified product. Labeled bacterial products were fragmented, biotin labeled, and hybridized to the PhyloChip Array, version G3. PhyloChip arrays were washed, stained, and scanned using a GeneArray scanner (Affymetrix). Each scan is captured using standard Affymetrix software (GeneChip Microarray Analysis Suite). For detailed references please see [23] and [29]. The raw PhyloChip output files are available for download (http://greengenes.secondgenome.com/downloads/phylochip_datasets).

### PhyloChip G3 data acquisition

PhyloChip G3 data processing was performed using Sinfonietta as described in [30,31]. Briefly, after pixel summarization of the fluorescence image, background subtraction, noise estimation and array scaling [23], array fluorescence intensity (FI) of each probe on each array was collected, background-subtracted and normalized to non-16S rRNA gene spike-in controls as explained previously [30,31]. From 93 hybridizations, 78,393 probes were deemed responsive in one or more samples where responsive was defined as described in [23]. In Sinfonietta, individual probes are clustered into probe-sets based on both 1) correlations in ranked FI across all samples and 2) taxonomic relatedness [24]. Then probe sets were annotated against GreenGenes lineages from phylum to species with a naïve Bayesian-algorithm-based classifier against the 2013 GreenGenes taxonomy [32]. Where standard taxonomic names were not available, a hierarchical taxon identifier was used (for example “94otu36152”). The clustering process contained a stochastic step when encountering ties in probe FI, therefore this step was repeated 5 times. The five runs resulted in an average of 1,579 probe-sets (SD = 1.85). The intersection of species-level lineages considering all five runs contained 883 different lineages. All probe-sets mapping to the 883 lineages were retained, totaling 1,491 probe-sets. The HybScore for each empirical OTU (eOTU) was calculated as the average ranked FI of the probes assigned to the probe-set. A binary score for eOTU was assigned with a 1 if > = 80% of the probes in that set were responsive in a given sample, otherwise a 0 was assigned (presence/absence estimation).

### Microbial community statistics

Microbial community analysis was performed using Second Genome’s PhyCA-Stats software package. In brief, relative abundances of eOTUs were rank-normalized across each array before applying any univariate or multivariate statistics. The weighted-UniFrac algorithm [33] was applied to calculate the pairwise sample dissimilarity, which was used to test for community relationships via ordinations (non-metric multi-dimensional scaling; NMDS) and Adonis (“permutational MANOVA”; [34,35]). In order to analyze community relationships for one sample type (N, C, D, S, T) the entire microbiome data was limited to the eOTUs called present in that particular sample type. Correlation analysis of eOTUs’ rank-normalized abundances and WGA treatment (preWGA = 0, lowWGA = 1, highWGA = 2) was calculated using Pearson’s linear correlation coefficient. A Welch test was applied to identify eOTUs that show a significant difference in their abundance between WGA treatments. Benjamini-Hochberg multiple testing correction was applied for all univariate analyses.

### Testing the effects of primer bias, 16S rRNA gene GC content, genomic GC content and genome size on microbiome structure

16S rRNA gene sequences were retrieved from the Greengenes database (version May 2013 [32]). The GC-content and a weighted primer score for each of the two primers 27F.1 and 1492R.jgi were calculated for each individual sequence using esl-seqstat from the HMMER package [36] and Primer Prospector [37]. Information on microbial genome size and genomic GC-content was extracted from the IMG database [38] for all prokaryotic genomes with status “Finished”, “Permanent Draft” or “Draft” (release January 2014). OTUs that were present both in Greengenes and IMG were determined based on a mapping file downloaded from Greengenes (S1 Table). The distribution of 16S rRNA gene GC-content, primer score, genome size and genomic GC-content was visualized for the taxonomic levels phylum, class, order, family, genus, and species. For each of these six taxonomic levels the correlation coefficients and p-values were calculated for all possible pairs of the four factors using Spearman’s rank correlation.

Correlation coefficients and p-values were calculated in a similar way for each of the four factors and the shift in the each of the eOTUs’ rank-normalized abundances between WGA treatments (preWGA to lowWGA, preWGA to highWGA and lowWGA to highWGA) individually for each sample type. The aggregated abundance values of one representative of each family that showed a significant difference in abundance between WGA treatments based on a Welch test were visualized on an idealized phylogenetic tree of the entire microbiome using iTOL [39]. For all OTUs classified at family level the 16S rRNA gene GC-content was also shown in the tree, as well as the genomic GC-content for OTUs having a representative in IMG database.

## Results

### Microbiome segregation by sample type was possible despite WGA treatment

**Fig 1:**
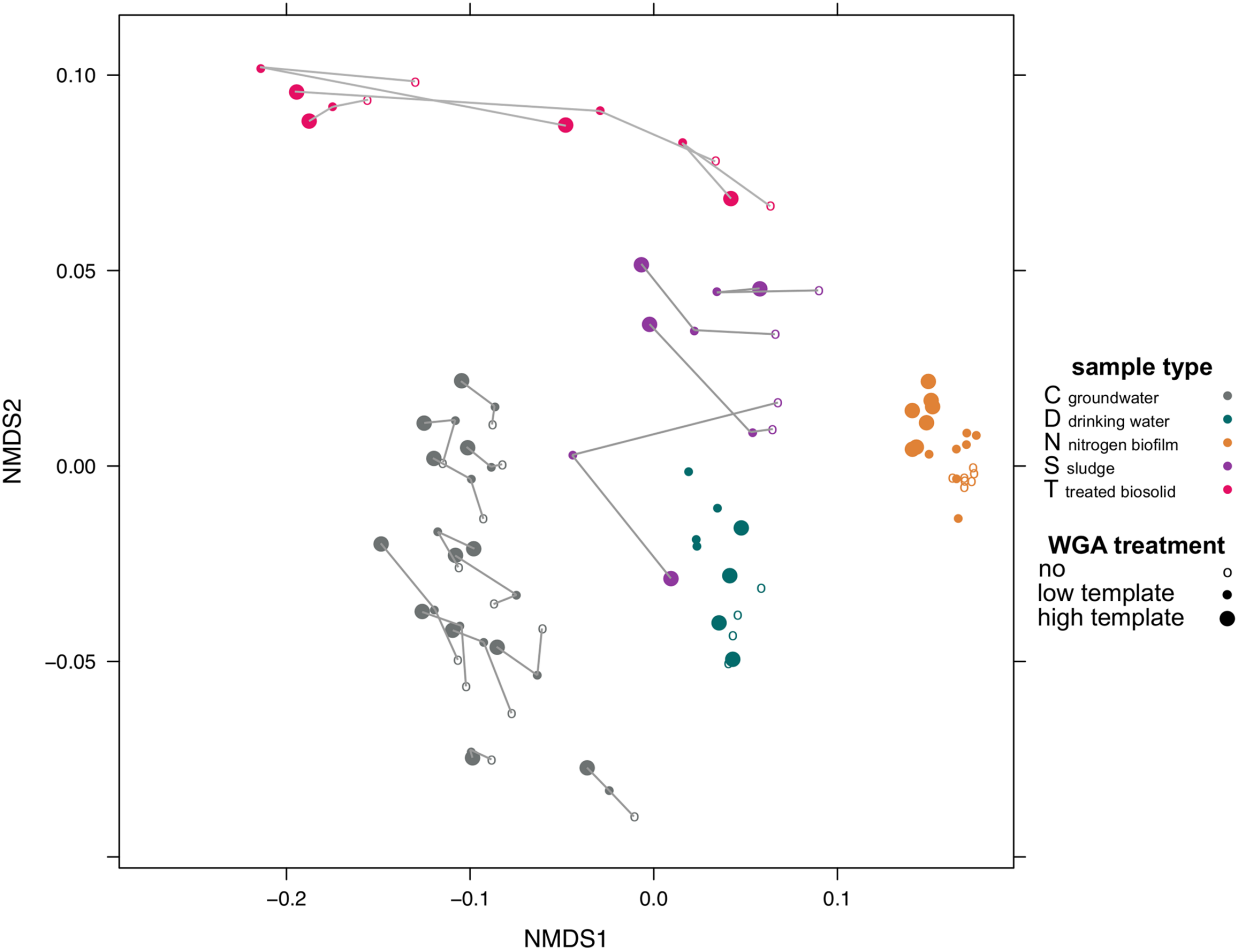
Various effects of WGA treatment of samples from five biotopes investigated by ordination analysis. NMDS analysis of whole community profile of 31 different samples from 5 different biotopes. All samples were either without treatment (“pre”), treated with “low” WGA (0.4 ng/μl) or with “high” WGA (4.0 ng/μl) resulting in 91 samples in total colored by biotope. For groundwater (C), sludge (S), and treated biosolid (T), the different WGA treatments of each individual sample are connected by grey lines (from “pre” to “low” to “high”). When a separation of samples based on the different WGA treatments was observed [biotopes drinking water (D) and nitrogen biofilms (N)], samples were not connected. Stress: 0.0983, number of OTUs: 1491.

Samples from five different biotopes were analyzed with respect to community differences before, after low and after high WGA treatment. These samples encompassed groundwater (C), drinking water (D), nitrogen biofilm (N), raw sewage sludge (S), and treated biosolids (T) and added up to 93 different samples (31 biological samples with pre, low and high WGA treatment). Based on the abundances of 1,491 eOTUs present in at least one of the samples, samples from different biotopes formed separate groups in ordination analysis (Fig 1). Although WGA treatment was applied to two third of the samples, differences in the microbial community relationship persisted across biotopes (Adonis p-value = 0.001) rather than across different WGA treatments (Adonis p-value = 0.206). All sample types could be clearly differentiated with regard to their biotope origin, with the exception of drinking water and sludge. The latter two sample types revealed some ambiguity due to one potential outlier sample that was treated with high WGA (Fig 1).

In other words, the overall microbiome revealed no separation of samples from pre- and post-WGA treatment. However, differences in the WGA treatment of samples were detected using Adonis testing for two out of five sample types, namely nitrogen biofilm (Adonis p-value = 0.001) and drinking water (Adonis p-value = 0.005), when considering the biotopes as individual sample sets. Here, a clear separation of pre, low and high WGA treated samples was observed (Fig 1). Groundwater samples showed a shift of the WGA treated microbiomes towards the negative along NMDS1 axis but samples with the same sample ID still grouped together (indicated by grey lines in Fig 1). Nevertheless, an entire separation of the C samples based on WGA treatment could not be captured (Adonis p-value = 0.354).

### WGA distortion was non-uniform across sample types

**Fig 2:**
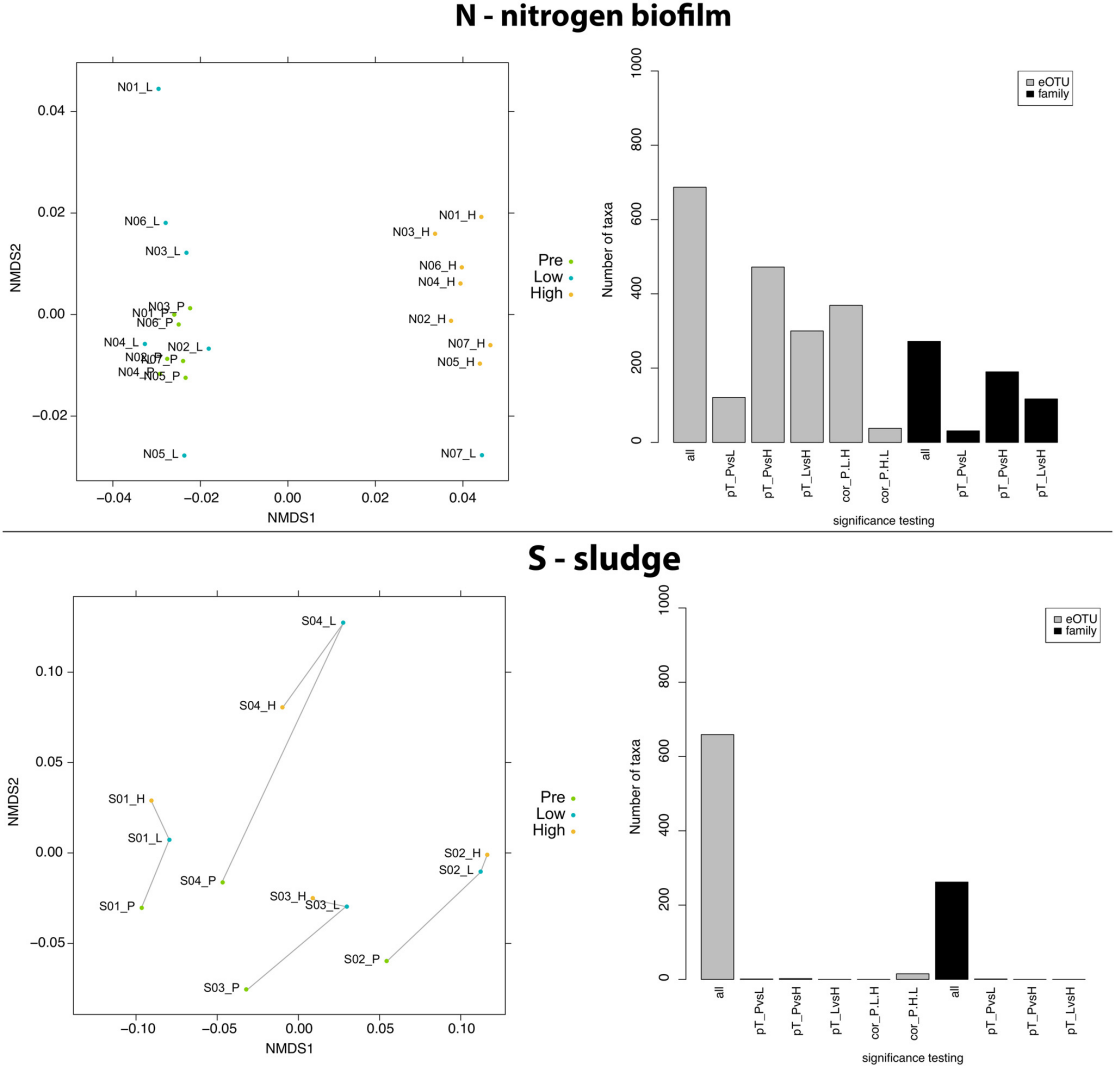
Individual analysis of samples from biotope nitrogen biofilm and sludge: NMDS (left panel) and differentiating taxa (right panel). N (nitrogen biofilm). NMDS analysis shows a separation of samples with high WGA treatment along NMDS1 axis (stress: 0.0628). Bargraph depicts the number of different taxa passing certain statistical tests. All test were corrected for false positives using the Benjamini-Hochberg correction. *all*: the total number of eOTUs considered for this analysis (called present in at least one of the N samples); *pT_PvsL*: Number of taxa that were significantly different between pre-WGA and low-WGA treatment using a paired t-test; *pT_PvsH*: Number of taxa that were significantly different between pre-WGA and high-WGA treatment using a paired t-test; *pT_LvsH*: Number of taxa that were significantly different between low-WGA and high-WGA treatment using a paired t-test; *cor_P*.*L*.*H*: Number of taxa that showed a significant correlation with the samples in the order pre-low-high using a Pearson correlation; *cor_P*.*H*.*L*: Number of taxa that showed a significant correlation with the samples in the order pre-high-low using a Pearson correlation. S (sludge). NMDS analysis shows a separation of samples with high WGA treatment along NMDS1 axis (stress: 0.0785). Bargraph depicts the number of different taxa passing certain statistical tests. All test were corrected for false positives using the Benjamini-Hochberg correction. Bargraph labels are according to N (see above).

In order to analyze the effects of WGA treatment on the microbial community and calculate the different levels of perturbation for each sample type, their microbiomes were separately investigated at eOTU and family level. First, we constrained the analysis of each biotope solely to those eOTUs called present in these samples. Ordination analysis calculated from these subsets did not reveal different intra-biotope microbiome relatedness compared to those presented in Fig 1 (Fig 2 and S1 Fig). The differences in the overall community structure of biotope N and S are depicted in Fig 2 as two examples where WGA treatment introduced a strong perturbation of the microbiome (biotope N) and where no significant influence of WGA treatment on the microbiome was observed (biotope S; other biotopes are displayed in S1 Fig). For sample type N, the greater the initial DNA concentration for WGA treatment, the greater was the perturbation to the perceived community.

With regard to WGA perturbation of single eOTUs or entire microbial families, a paired t-test and a correlation analysis coupled to a Benjamini-Hochberg correction for false discovery were performed (Table 1). While biotopes C, D, and N revealed a high proportion of distorted eOTUs and families, samples of the type S and T revealed marginal to no effect due to WGA treatment (Fig 2 and S1 Fig).

**Table 1:** Significant taxa per biotope.

taxonomic level	biotope	C	D	N	S	T
eOTU	total number of eOTUs	879	461	687	659	603
	paired t-test: pre versus low	165	185	121	1	1
	paired t-test: pre versus high	256	8	472	2	0
	paired t-test: low versus high	27	1	300	0	0
	correlation: pre—low—high	19	9	369	0	0
	correlation: pre—high—low	24	174	38	15	7
family	total number of families	305	180	272	262	287
	paired t-test: pre versus low	52	49	31	1	0
	paired t-test: pre versus high	102	1	190	0	0
	paired t-test: low versus high	4	2	117	0	0

Number of taxa passing certain statistical tests when comparing samples of before, after low and after high WGA treatment. The analyses were carried out at eOTU and at family level. For the latter, abundances of eOTUs were aggregated. Please see Fig 2 and S1 Fig for graphical representation of the data.

### GC content was significantly correlated with WGA perturbation while primer affinity score and genome size were not

**Fig 3:**
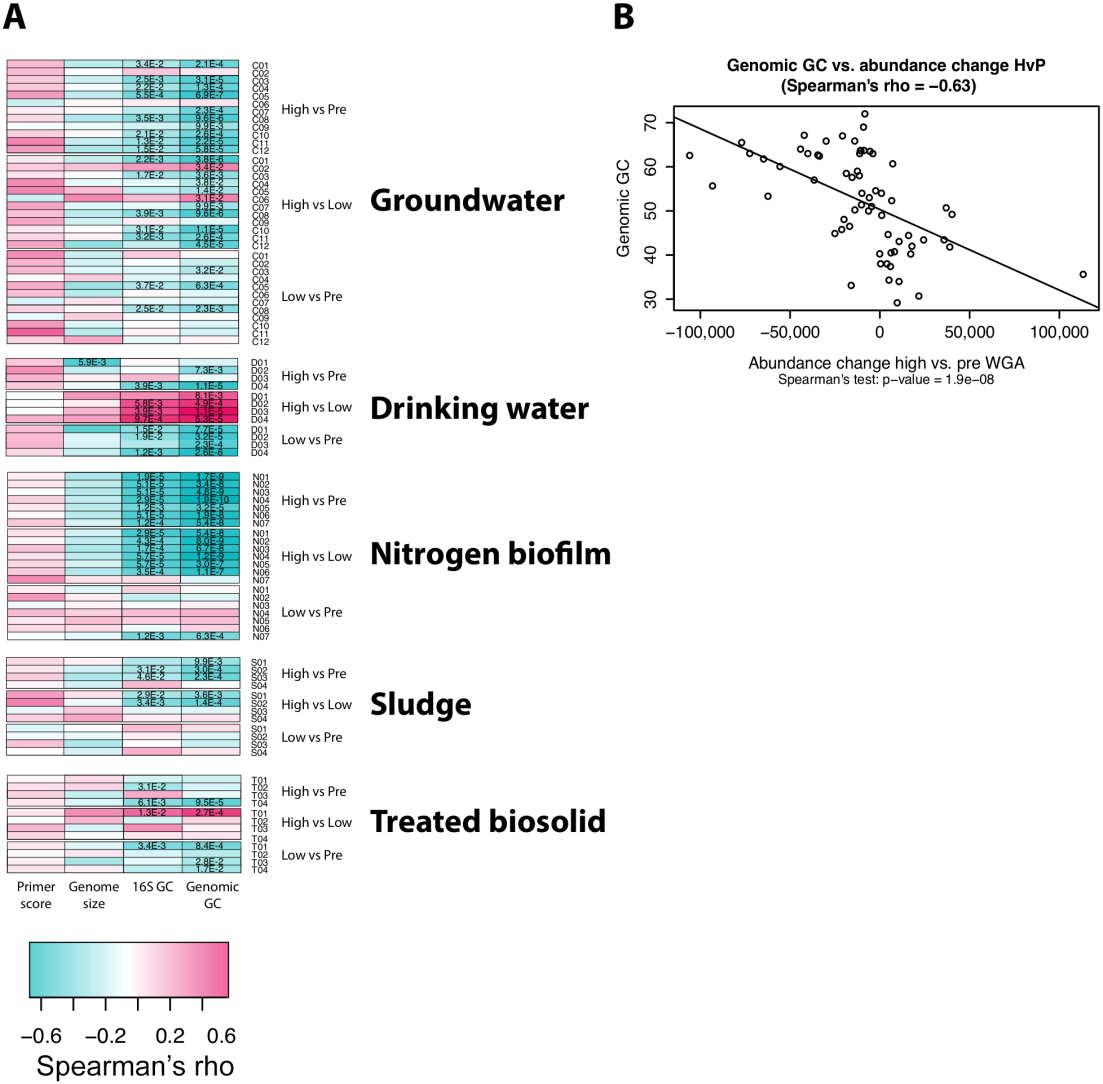
Correlation analysis between OTU abundance shift and genomic properties on family level. A: Heatmap of correlation coefficients (Spearman’s rho) between the OTU abundance shift and the mean per family of four genomic properties. Each row contains the correlation coefficients for OTU abundance shifts between two WGA treatments (high vs. pre, high vs. low or low vs. pre) for one sample. Each column shows the correlation coefficients for one genomic property (Primer score, Genome size, 16S GC and Genomic GC). Dark turquoise color indicates a negative correlation, dark red color a positive correlation. Significant p-values are given as numbers in each cell after applying Benjamini-Hochberg correction. B: As an example of the correlation of genomic GC and abundance changes, significantly different families for sample type N (pre vs. hi, Fig 2 and S2 Fig) were used. The plot shows a significant negative correlation, as do all other analyses of the samples for significantly different families.

GC content has been hypothesized to be the major cause for WGA distortion in microbial communities but has only been analyzed for one microbial community of high biomass [22]. Here, we analyzed low and high biomass samples and investigated the effect of WGA on four intrinsic genomic properties by correlating them individually with OTU abundance shifts on family level. The considered properties were the genome size and the genomic GC content as well as two other factors to biases arising from 16S amplicon analyses, namely GC content of the 16S rRNA gene and “PCR-ability” of the 16S rRNA gene sequence represented by its primer affinity score. The distributions of these properties across all taxonomic levels including all taxa present in the IMG database (considering only families with representative genomes) are depicted in S2 Fig We calculated the Spearman’s correlation coefficient between the mean abundance shift (pre versus low, pre versus high and low versus high) per family and the mean of the genomic property for this family for every sample (Fig 3A).

Considering the entire sample set, there was no significant correlation for the two genomic properties primer score and genome size, with regard to abundance shifts of the observed families (exception for one drinking water sample pair that showed a significant correlation to genome size). However, 42 and 55 out of 93 samples showed a significant correlation (p<0.05) for 16S rRNA gene GC-content and genomic GC-content, respectively. Overall the 16S rRNA gene GC-content had a highly significant positive correlation with genomic GC content (pair-wise correlations of the four properties tested are presented in S3 Fig). Therefore, genomic GC-content was considered a driving force in the observed WGA treatment perturbation among the four genomic properties considered.

Importantly, given the significant correlations of differences in relative abundances of families with genomic GC content, both low and high WGA treatment versus pre WGA treatment consistently resulted in a negative correlation irrespective of the sample type analyzed. However, high versus low WGA treatment showed ambiguity across the sample types concerning positive and negative correlations. For instance sample type D revealed only positive correlations, while sample type N constantly negative correlations. Sample type C showed mixed responses for high versus low.

Taken together, from the constant negative correlation of genomic GC content with abundance scores in WGA treatment versus no WGA treatment (high versus pre and low versus pre) can be concluded that genomes with low GC-content showed an increase in abundance after WGA treatment, while genomes with high GC-content showed a decrease in abundance after WGA treatment. This WGA bias is particularly strong for families with significantly different abundance changes (example is depicted in Fig 3B). Across all samples, only negative correlations were detected for families with significantly different abundance changes.

### WGA treatment decreased perceived abundance of Alpha- and Betaproteobacteria

Considering the change of abundances in each family detected in the sample sets, the perturbation of the community before and after WGA treatment varied greatly (Table 1). For instance, high biomass samples such as T (treated biosolids) did not reveal any family with a significant microbiome change while sample type S (sludge) revealed only 1. These results are reflected by the ordination depicted in Fig 1, where S and T samples showed high microbiome variations across samples and a stochastic distribution of the microbiome relatedness could be hypothesized.

**Fig 4:**
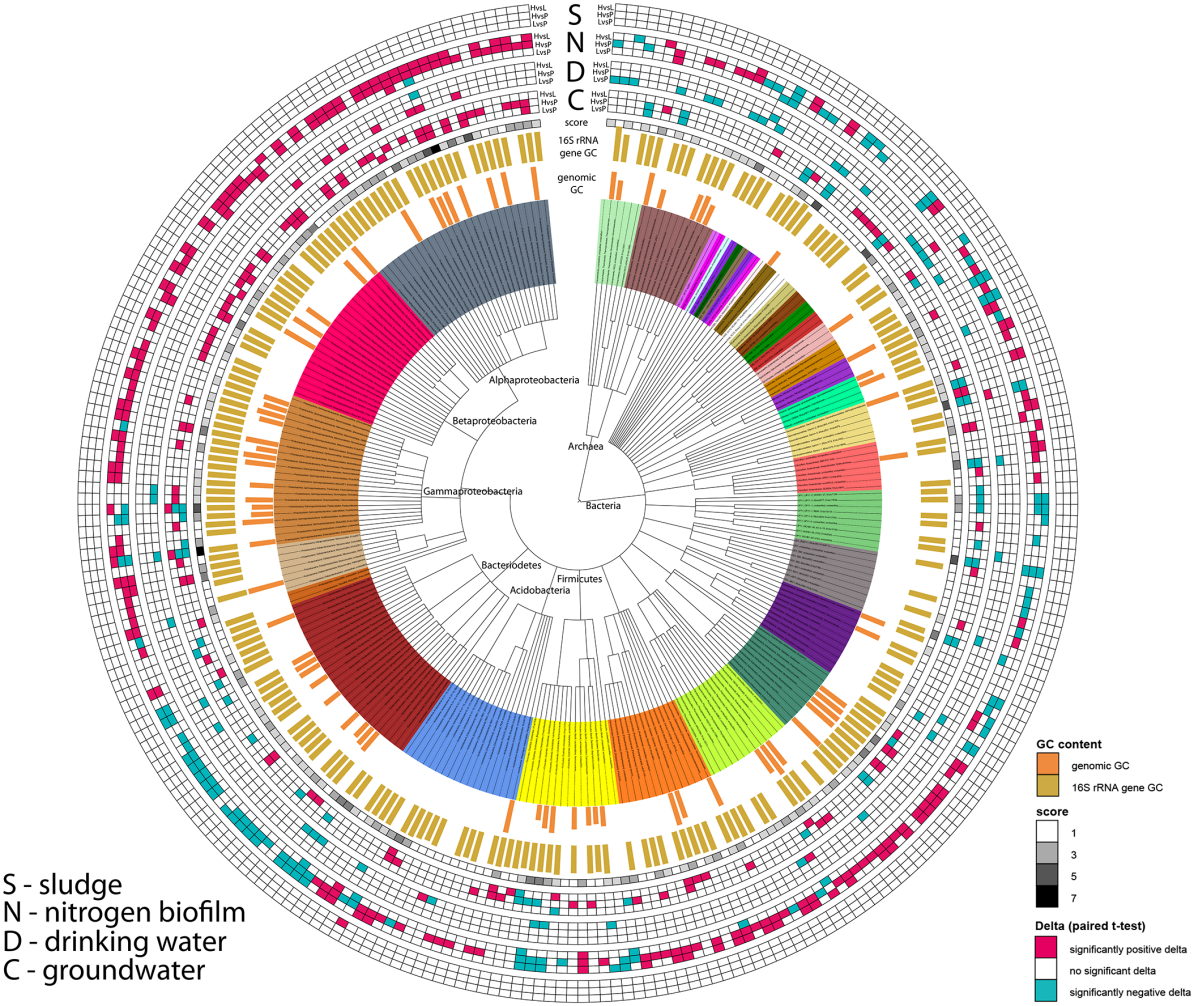
Taxonomic distribution of abundance shifts. Phylogenetic tree of families with significant abundances changes in pre versus low, pre versus high or low versus high WGA treatment samples (paired student’s t-test). Please note that sample type T (treated biosolid) did not reveal any families with significant changes. Dark blue indicates a significantly negative delta, while dark red indicates a significantly positive delta. The amount of significant t-tests per family is displayed in the ring “score”, whereas dark grey indicates 7 out of 15 tests were significant, while white indicates only 1 difference (families without significant changes are not displayed). The inner rings show barplots for 16S rRNA gene GC content and genomic GC content.

In contrast to the high biomass samples (S and T), the low biomass samples (N, D and C) had many taxa that showed a systematic change in their abundance due to the WGA treatment (Table 1, Fig 4). Among these taxa, the dominant lineages were Alpha- and Betaproteobacteria (Fig 4). All taxa with a significant change in their abundance in these classes showed a positive delta, meaning that their abundance decreased after WGA treatment (one exception: unclassified family in the Sphingomonadales, Alphaproteobacteria). These same families with representative genomes in the IMG database in our samples had a relatively high average genomic GC-content (>53%, max. 67%), which is in accordance with the results presented in Fig 3, with both classes underrepresented after WGA treatment, most likely due to their high genomic GC content.

### Enterobacteriaceae showed the strongest perturbation after WGA treatment

The effect of WGA treatment on the perturbation of each family was captured by scoring significant p-values across all samples. For the five sample types, three paired t-tests on abundances of families were computed, resulting in a maximum score of 15 across all samples. The results were, however, not only affected by the significant change of a taxon in its abundance but also by its presence in a particular dataset. Consequently, the two families that showed the most number of significant changes (7) were unclassified *Sphingomondales* (Alphaproteobacteria) and *Enterobacteriaceae* (Gammaproteobacteria; Fig 4, inner ring with grey scaling). Both of these families did not follow a uniform pattern, meaning their abundances were up and down regulated by WGA treatment. Nevertheless, the major abundance differences of all families analyzed were identified for the *Enterobacteriaceae*, which showed the strongest perturbation due to WGA treatment based on abundances and statistical binary scoring.

## Discussion

Understanding the bio-chemical cycling within ecosystems generally starts with identifying the diversity of functional genes within its microbial consortia but only 0.1% of all microbial taxa may be cultivated and studied in the laboratory [1,2]. Multiple lineages tracked in this study (~50% on family level) have no genomes yet sequenced underscoring the need to isolate a more diverse set of microbes for environmental microbiology. To overcome cultivation limitations, environmental genomics has become an attractive alternative to cultivation in order to propose metabolic pathways existent in less understood biotopes [40]. However, retrieving sufficient biomass to perform next-generation sequencing of metagenomic DNA is sometimes hampering these processes, forcing scientist to use whole genome amplification (WGA) methods to gain “something from (almost) nothing” [41]. Previous work, investigating the effects of WGA on natural microbial communities has either focused on samples from one biotope [17,18], and/or did not include samples from low biomass sites [21,22]. The conclusions that can be drawn from the literature are obscured by the various 16S rRNA gene-profiling methods used for investigating the effects of WGA.

Using a highly reproducible microarray approach [23] across five different ecosystem biotopes revealed that WGA treatment did affect all sample types, since no sample community profile was the same before and after WGA treatment. However, the microbiome changes were not uniform across samples types. Raw sludge and treated biosolids samples revealed a stochastic bias; their microbiome change did not follow a reproducible and systematic manner considering biological replicates. Rather, microbiome differences after WGA treatment were larger compared to other sample types and future investigations may necessitate further analyses of the general reproducibility of the microbiome profile that can be retrieved from these biotopes. The effects of WGA treatment were not fully understood for these sludge and biosolid samples, and provides a warning to others working with similar material.

Of the sample types tested in this study where WGA is more likely to be used (e.g., groundwater, drinking water and nitrogen biofilm), however, there appeared to be a systematic bias, which was reproducible across biological replicates and demonstrated consistent abundance changes with genomic properties. The potential of an amplification bias during 16S rRNA gene PCR was ruled out, as primer affinity did not correlate with the observed abundance changes of families (Fig 3). While genome size did not correlate with abundance changes, both genomic GC content and 16S rRNA gene GC content did. It can be assumed that the genomic GC content is similarly affected as the 16S rRNA gene GC content, since these two variables show high correlation within one species as demonstrated (S3 Fig). These results were based on the genomic properties of microbial lineages that have a genome sequence available in public databases. Although 573 OTUs out of 1,491 OTUs could not be classified at family level and 201 OTUs with classifications did not have a representative genome sequence in public databases, our results are grounded on genome sequences of 112 different microbial families that span approximately 50% of the entire microbiome discovered (717 OTUs). However, metagenomic approaches, sometimes based on WGA, are nowadays able to reveal near complete to complete genomes [6,9] and may provide even more genomes in near future for verification of the results presented herein.

In conclusion, the genomic GC content was apparently identified as the major effector in WGA bias, although other variables like genome fragmentation could also potentially impact WGA changes [22]. A negative correlation between GC content and post-WGA abundance has recently been documented for samples from one biotope [22], yet our results indicate that some sample types demonstrate a larger bias than others, and that high GC content genomes are not always preferentially amplified. Nevertheless, in most cases high genomic GC was negatively correlated with abundance changes, meaning that genomes with high GC content had lower abundances after WGA treatment and vice versa. Reasons for this may be the usage of random hexamers without high GC content hampering the amplification of GC-rich regions, although hexamer composition is proprietary, thus no further information is provided by the supplier.

Assuming high GC content leads to a lower representation of microbial lineages in samples after WGA treatment, there is potential to estimate the effects of WGA in certain sample types. Therefore, performing 16S rRNA gene profiling and comparing the retrieved lineages with genomes in public databases could help to estimate what types of microbial taxa are most problematic with respect to WGA. However, one family, Enterobactericeae, a key group used to represent enteric members, consistently demonstrated a major change (based on abundance delta and number of significant changes) across all sample types. This is of particular interest for drinking and in groundwater samples as members of this family have been associated with enteric waterborne diseases [42,43]. As one of the *Enterobactericeae* species (i.e., *Escherichia coli*) is used as a fecal contamination indicator [44], these results are also relevant to recreational waters and microbial water quality studies. WGA treatment of drinking water and groundwater may reveal strongly biased results either over- or underestimating the abundance of enteric bacteria, for which certain members require genome sequencing to separate environmental forms from true enteric members [45,46].

In sum, our results suggest that differences in microbiome relationships across different biotopes can be captured even after WGA treatment. However, intra-biotope relationships, i.e. samples with similar microbiome compositions, can be perturbed sometimes in an unpredictable manner no longer enabling the detection of abundances changes of microbial taxa across samples. For many samples (but not all), genomic GC content was significantly correlated with family abundance changes after WGA treatment: whereas high GC-genomes mostly decreased in abundance, low-GC genomes increased. The application of WGA treatment in research fields performing environmental genomics of hospitals, indoor microbiomes, air samples, clean room monitoring, and human skin samples could strongly benefit from this technology. Nevertheless, WGA treatment introduces biases that may hamper individual abundance change calculations of microbial taxa and necessitates monitoring via 16S rRNA gene amplicon sequencing.

## Supporting Information

S1 Fig Individual analysis of samples from biotope groundwater, drinking water and treated biosolid: NMDS (left panel) and differentiating taxa (right panel). C (groundwater). NMDS analysis shows a separation of samples with high WGA treatment along NMDS1 axis (stress: 0.0808). D (drinking water). NMDS analysis shows a separation of samples with high WGA treatment along NMDS1 axis (stress: 0.0668). T (treated biosolid). NMDS analysis shows a separation of samples with high WGA treatment along NMDS1 axis (stress: 1e-04). Bargraphs depict the number of different taxa passing certain statistical tests. All test were corrected for false positives using the Benjamini-Hochberg correction. Bargraph labels are according to Fig 2 (see above).
(PDF)

S2 Fig Compilation of distributions of genomic properties (Primer score, Genome size, 16S GC and Genomic GC) per taxonomic level. (PDF)

S3 Fig Compilation of correlation analyses of genomic properties (Primer score, Genome size, 16S GC and Genomic GC) per taxonomic level. (PDF)

S1 Table List of all OTUs that were used for S2 and S3 Figs along with their corresponding accessions in Greengenes, IMG and RefSeq and their NCBI taxonomy ID. If no ID was available for an OTU, “-1”is given.
(CSV)
