# Supplementary figures and images for: New Perspectives on Microbial Community Distortion after Whole-Genome Amplification
Source: PLoS One. 2015 May 26;10(5):e0124158. doi: 10.1371/journal.pone.0124158 (PMC4444113; doi:10.1371/journal.pone.0124158)

## C - groundwater

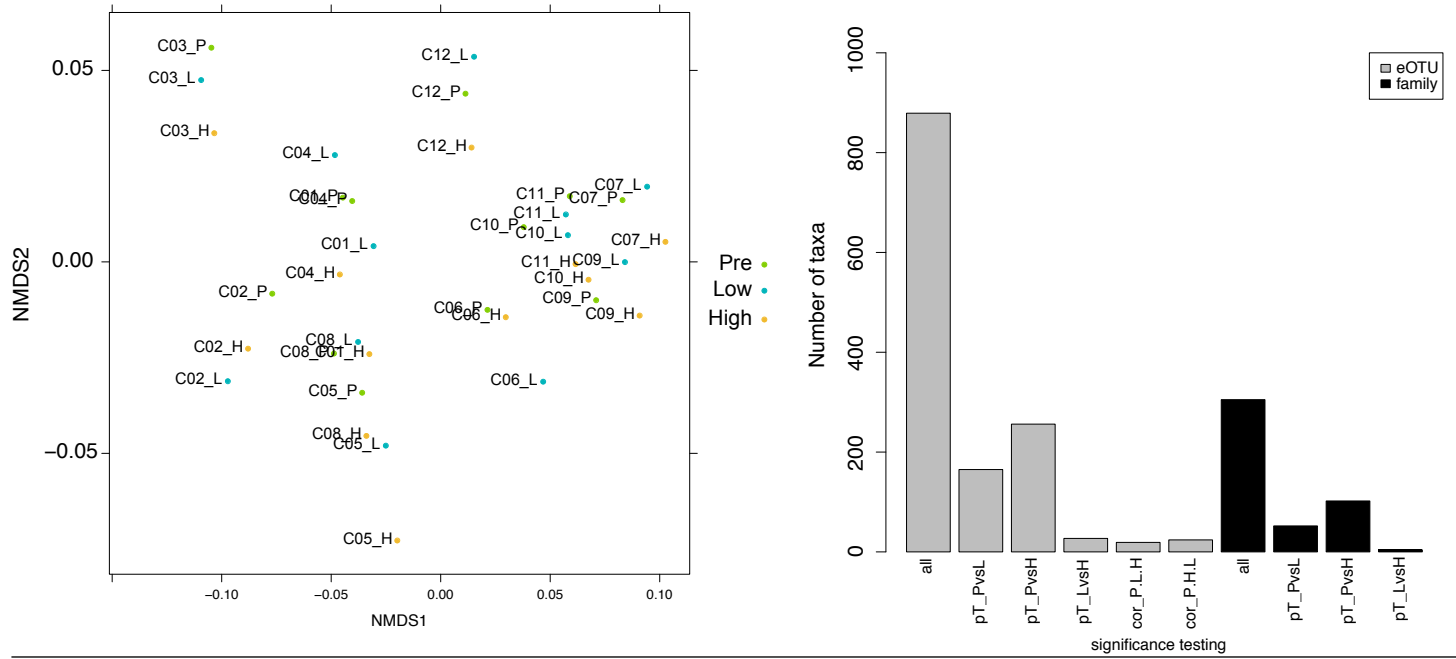

## D - drinking water

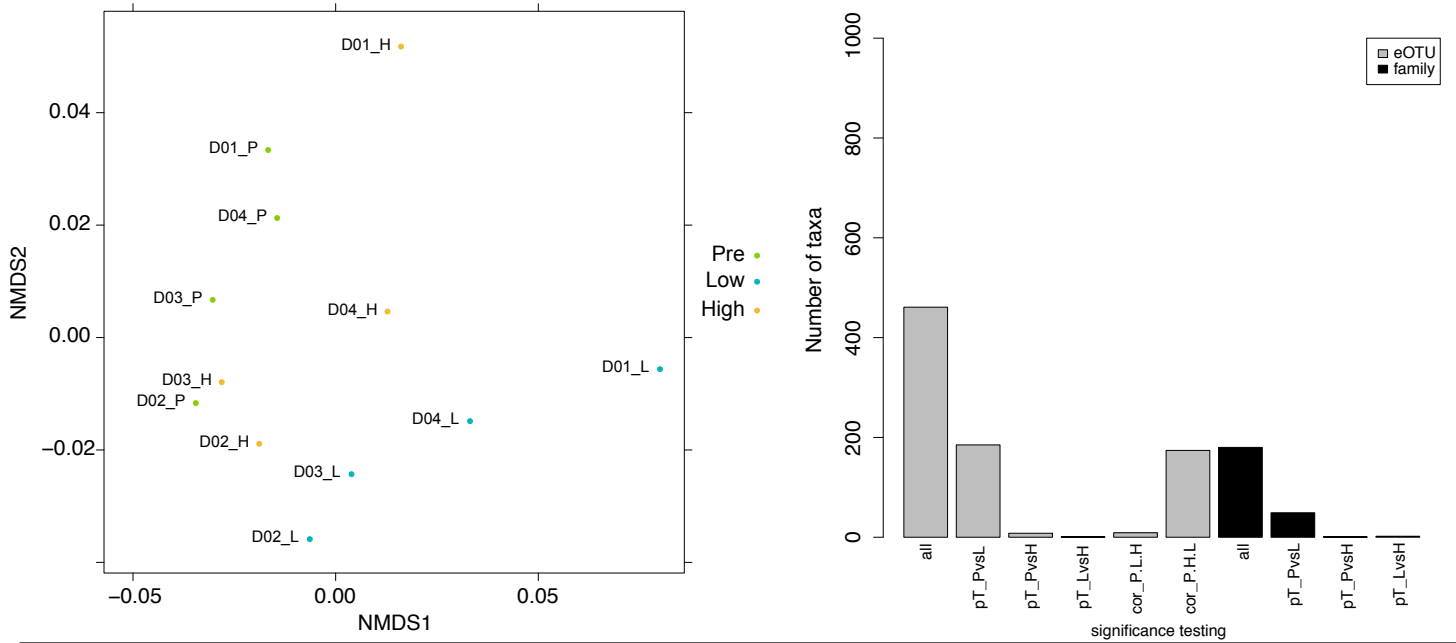

## T - treated biosolid

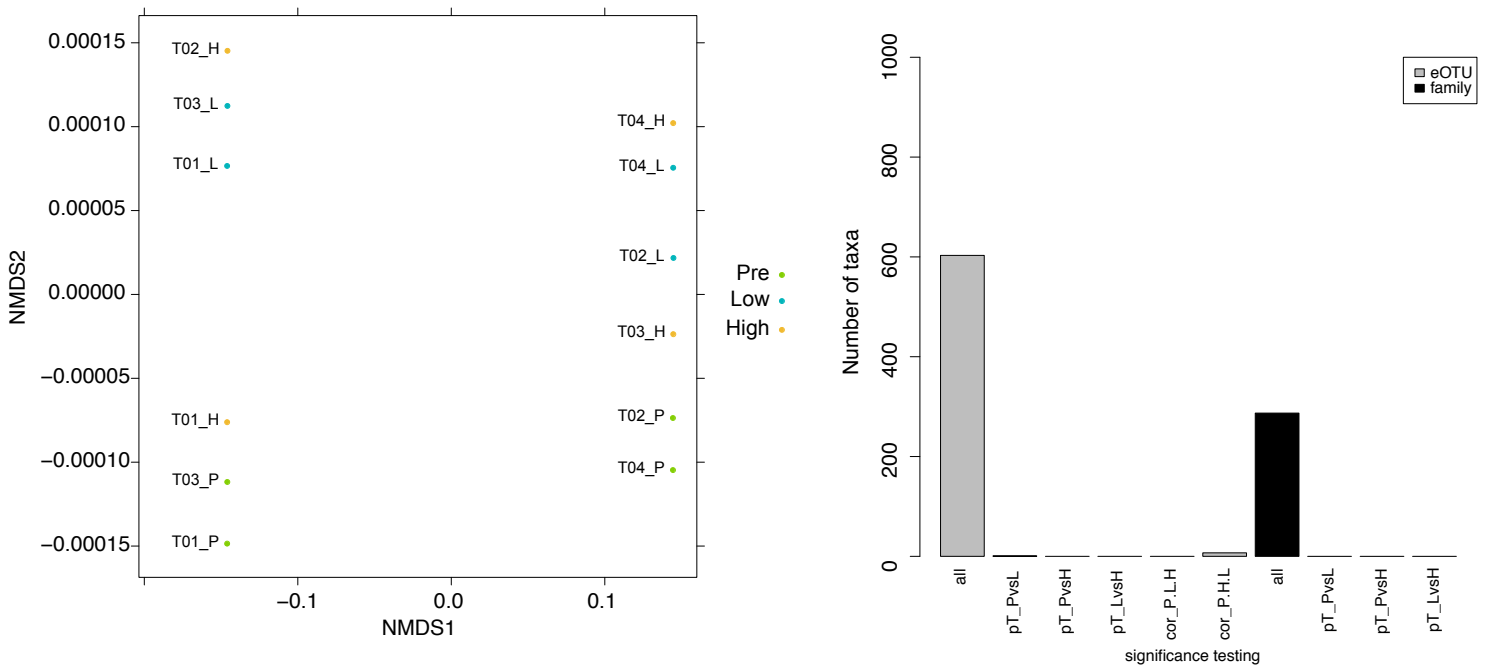

Supplement: S1 Fig — C (groundwater). NMDS analysis shows a separation of samples with high WGA treatment along NMDS1 axis (stress: 0.0808). D (drinking water). NMDS analysis shows a separation of samples with high WGA treatment along NMDS1 axis (stress: 0.0668). T (treated biosolid). NMDS analysis shows a separation of samples with high WGA treatment along NMDS1 axis (stress: 1e-04). Bargraphs depict the number of different taxa passing certain statistical tests. All test were corrected for false positives using the Benjamini-Hochberg correction. Bargraph labels are according to Fig 2 (see above). (PDF) [file pone.0124158.s001.pdf]

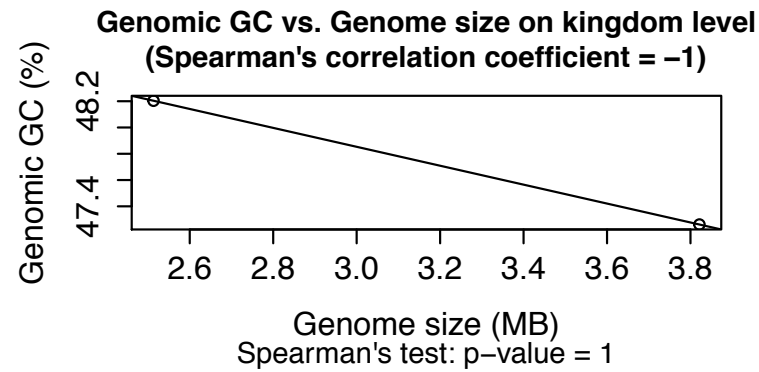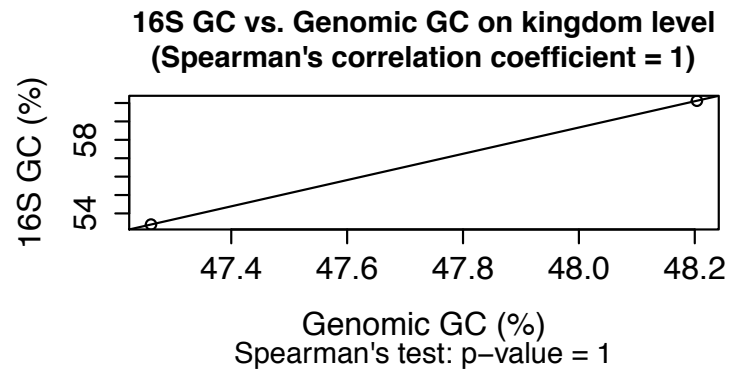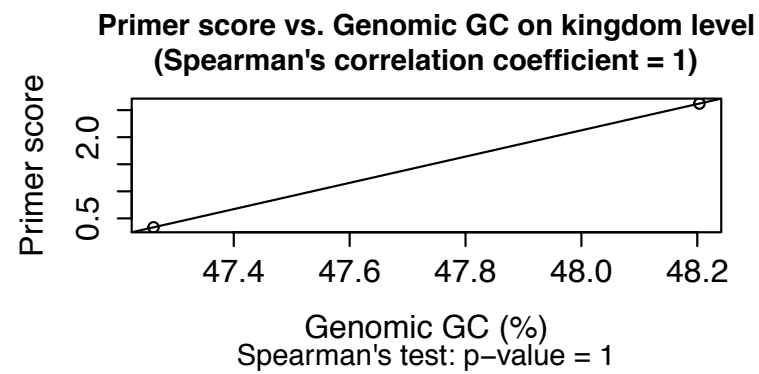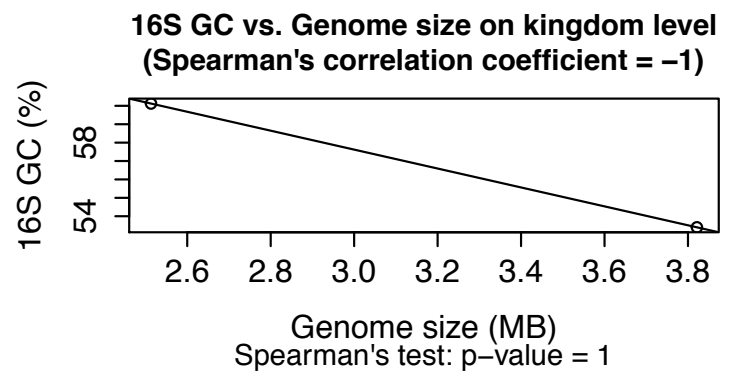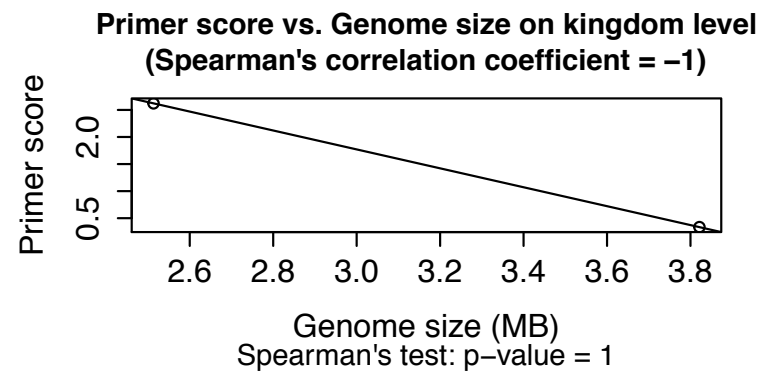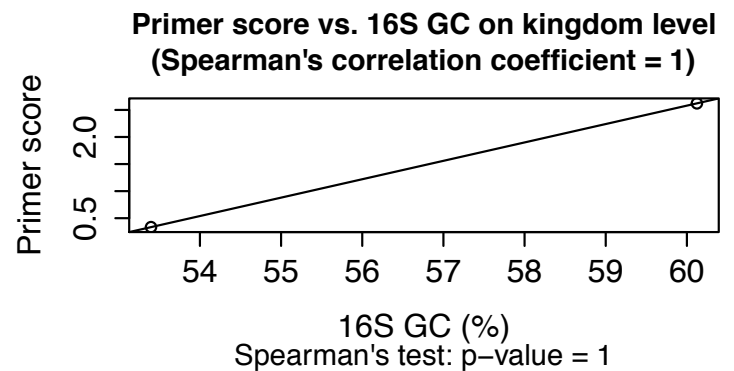

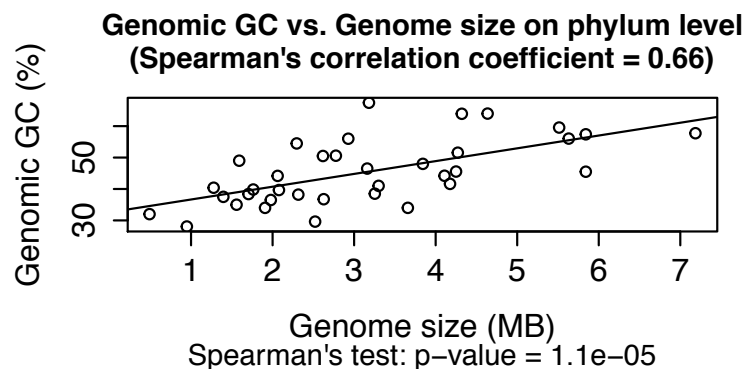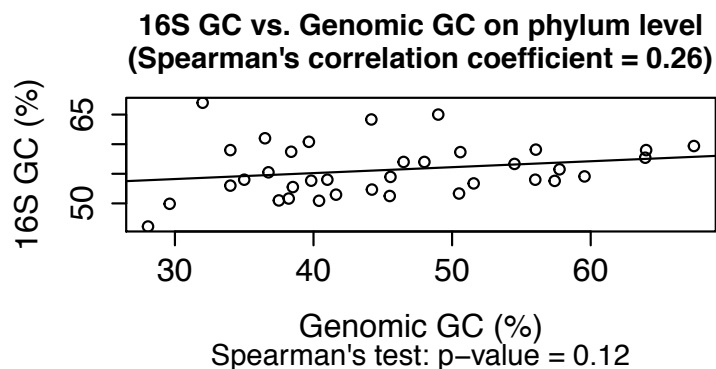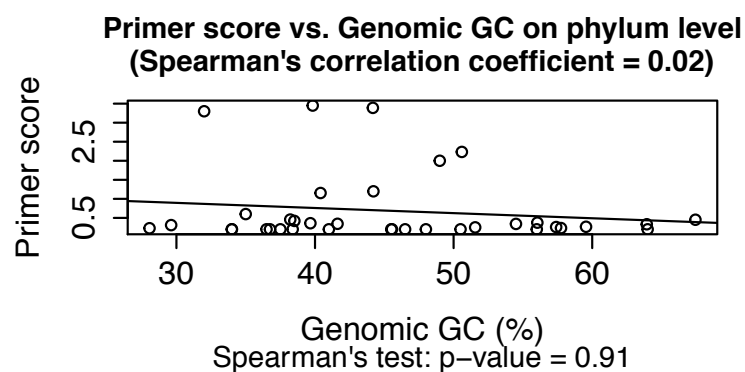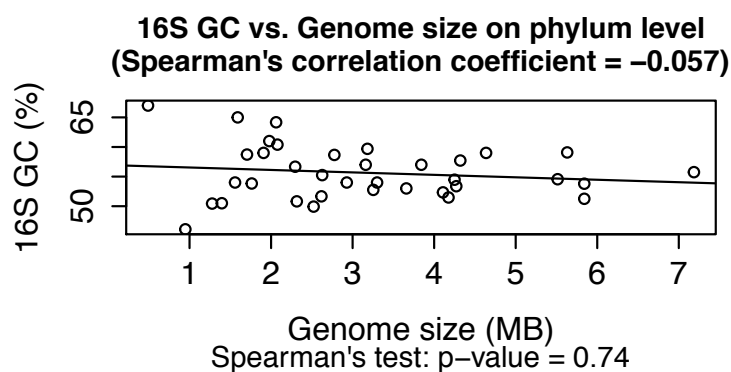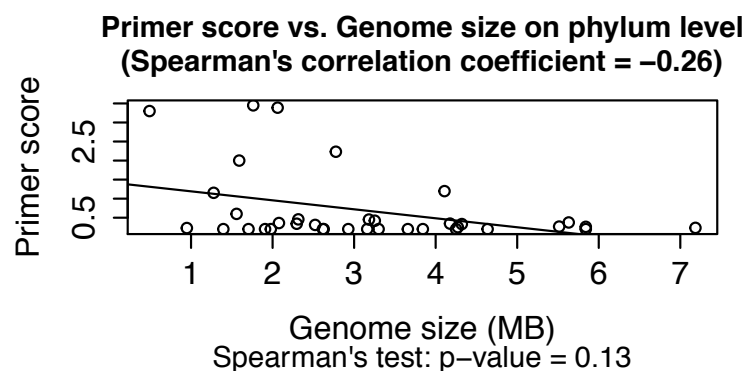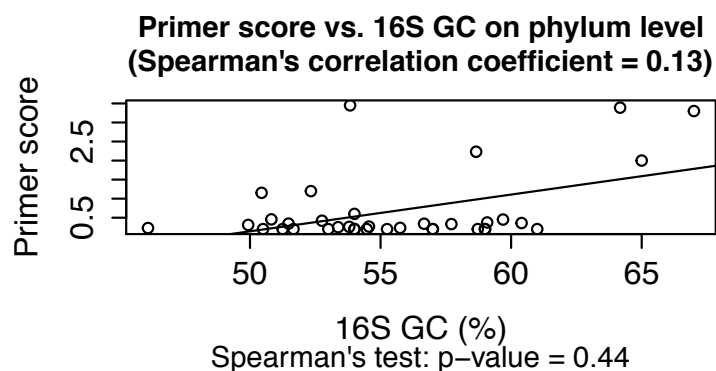

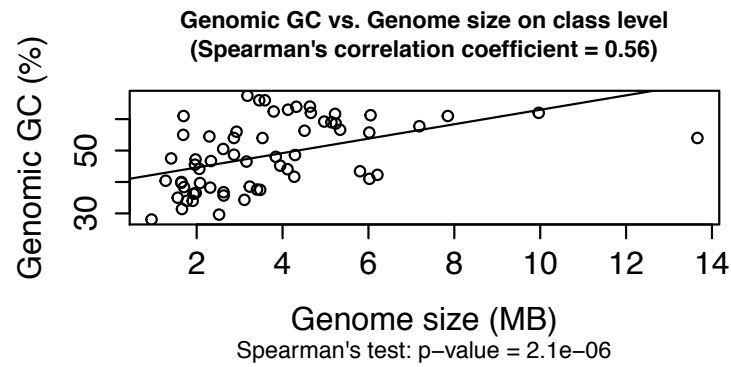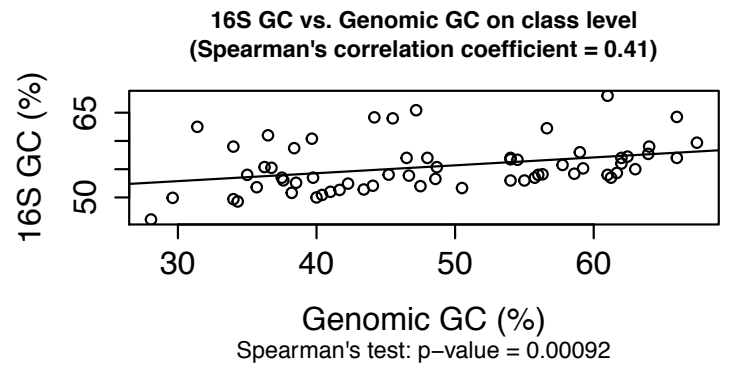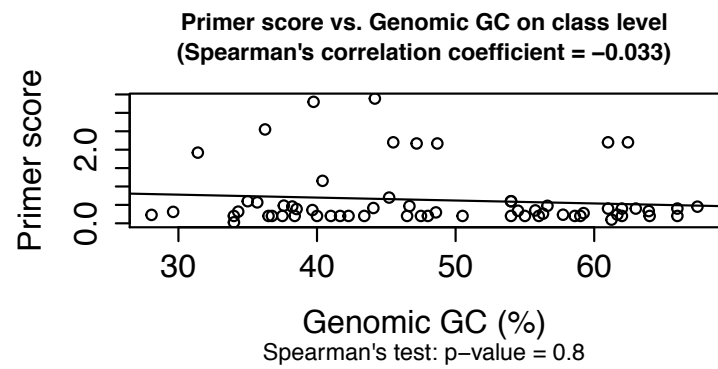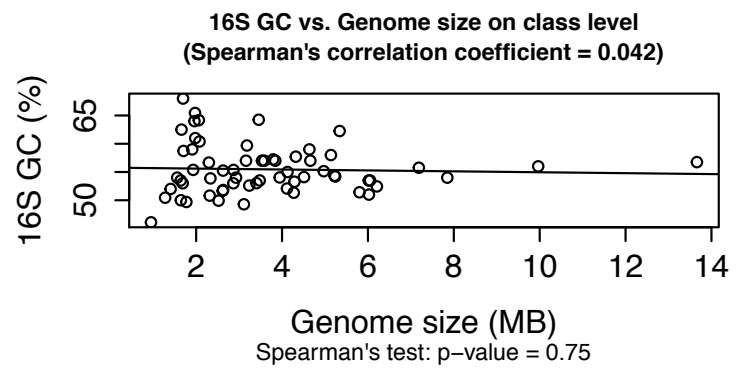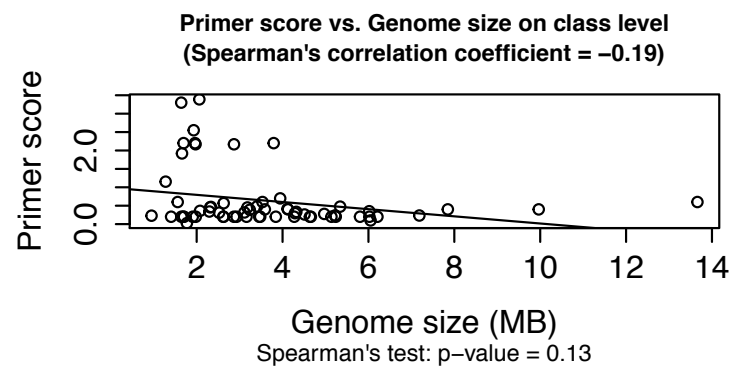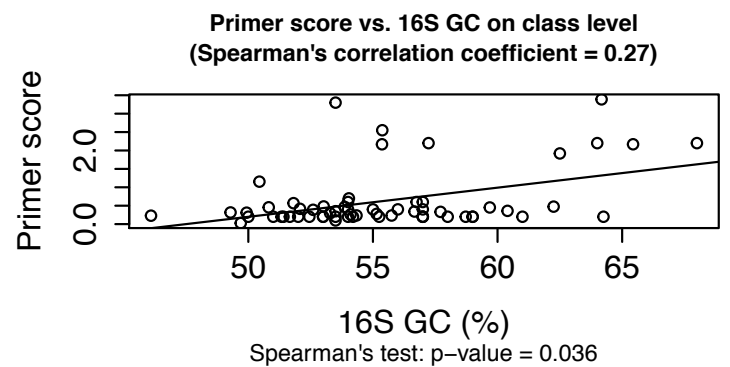

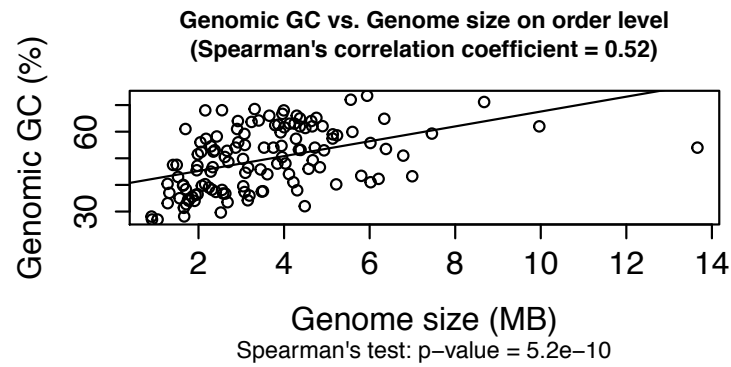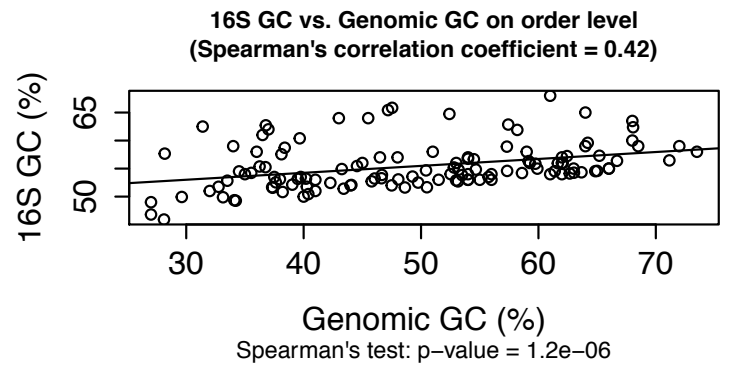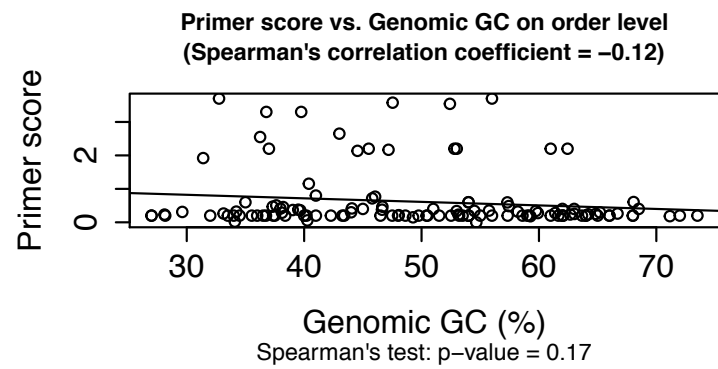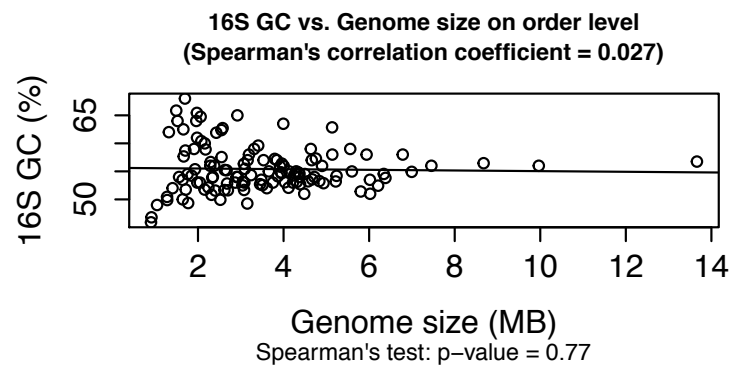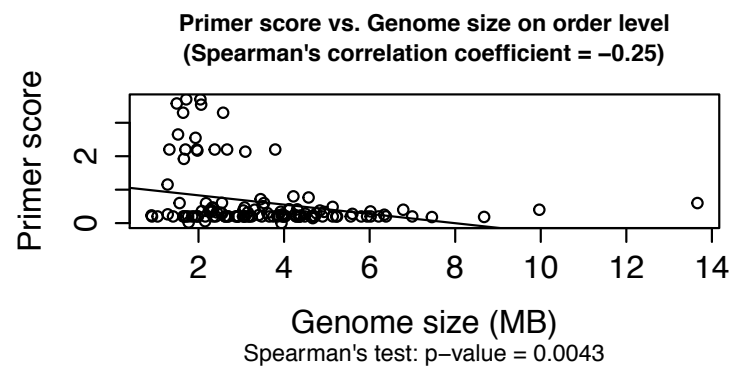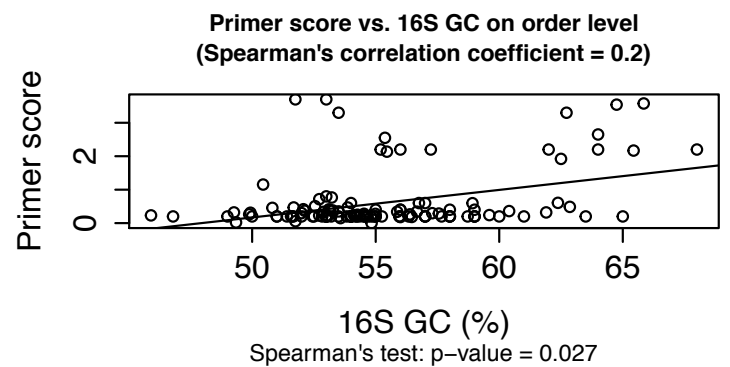

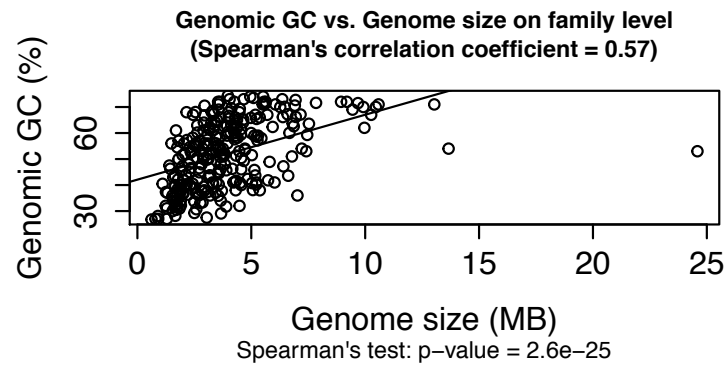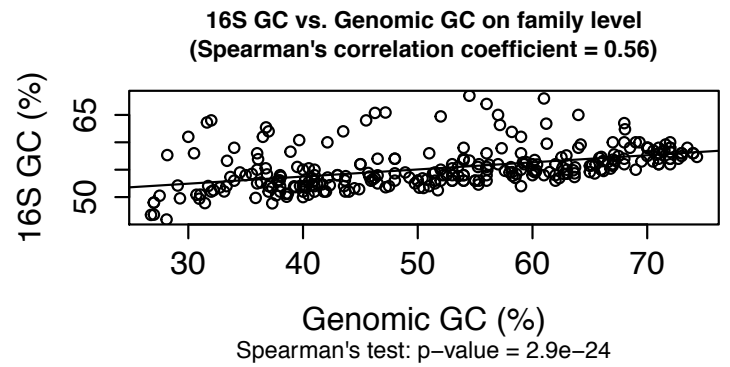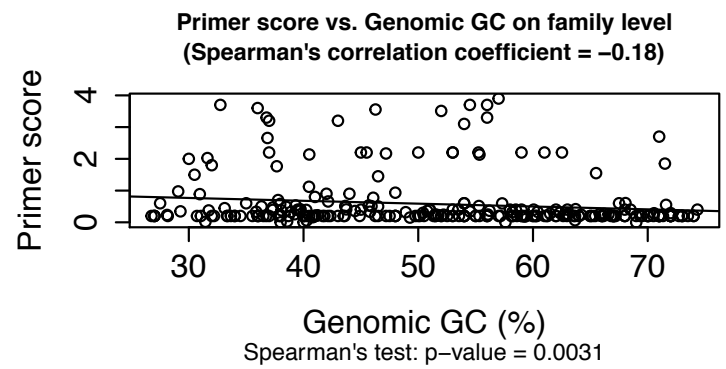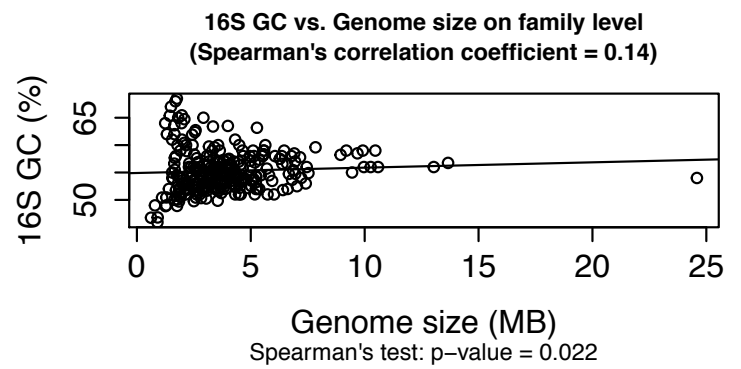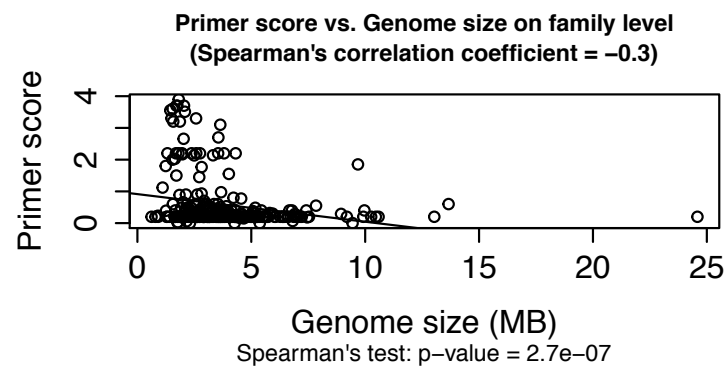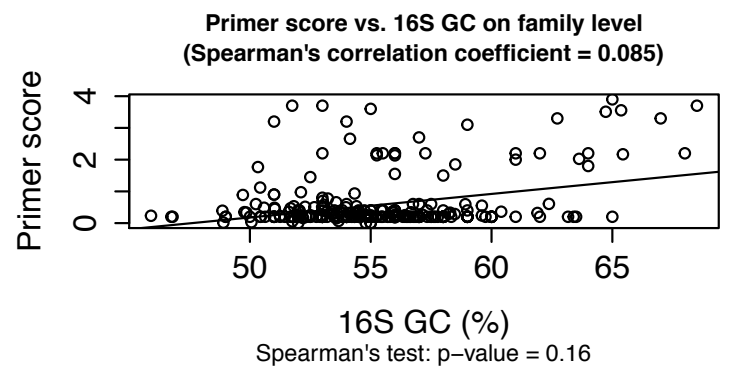

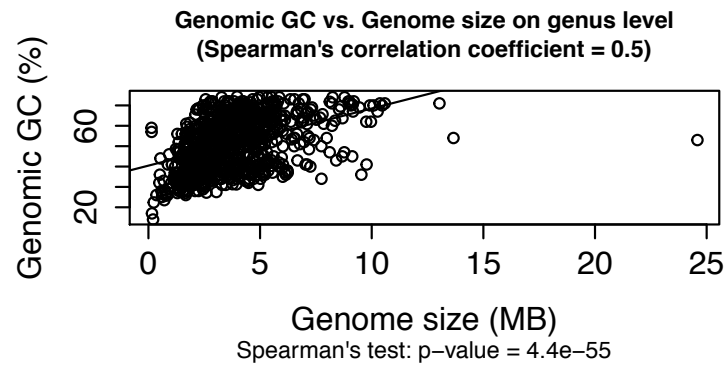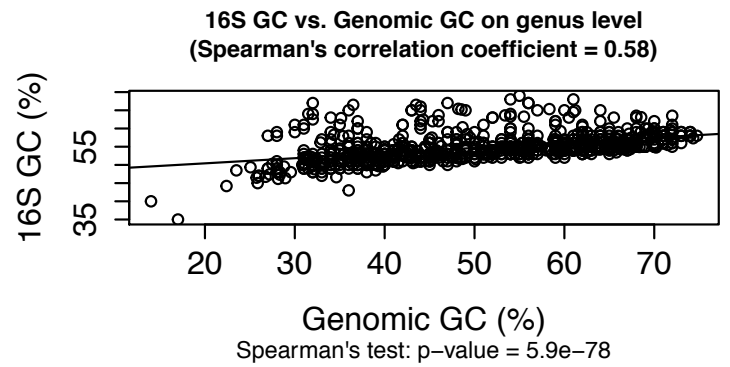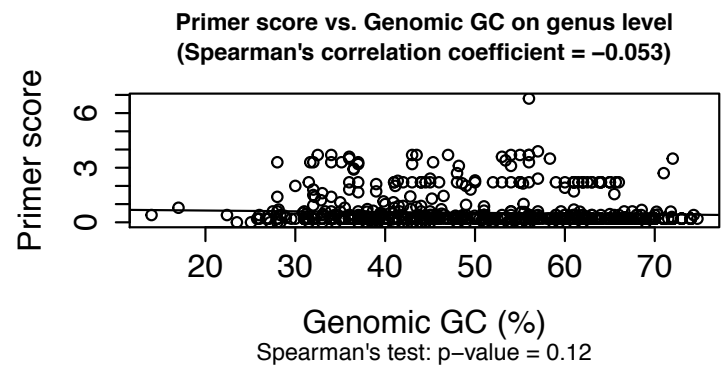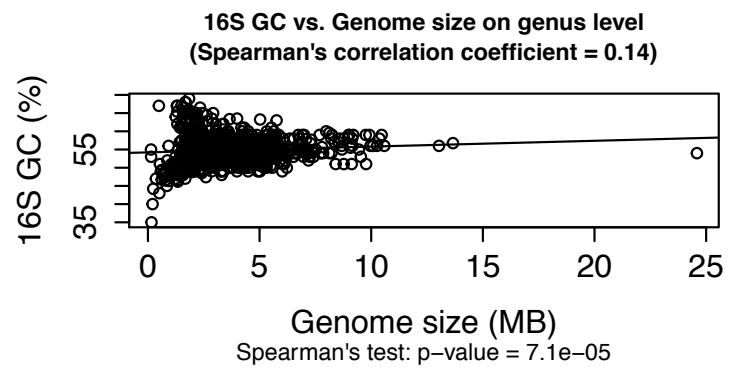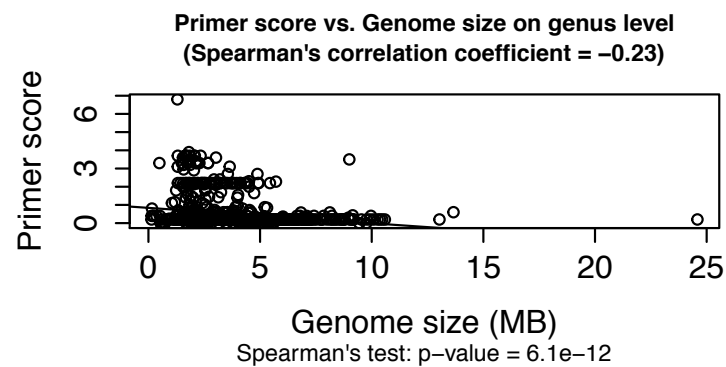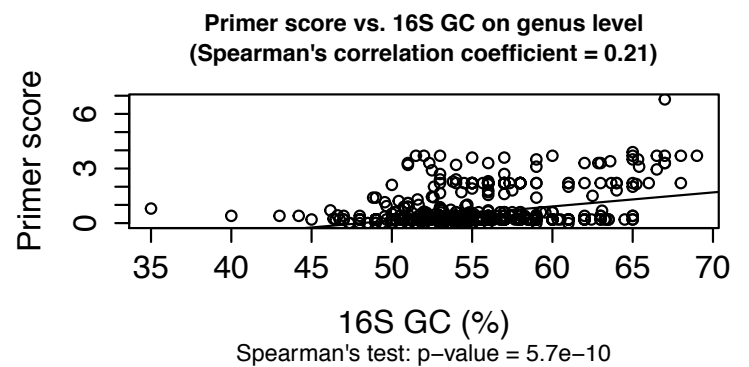

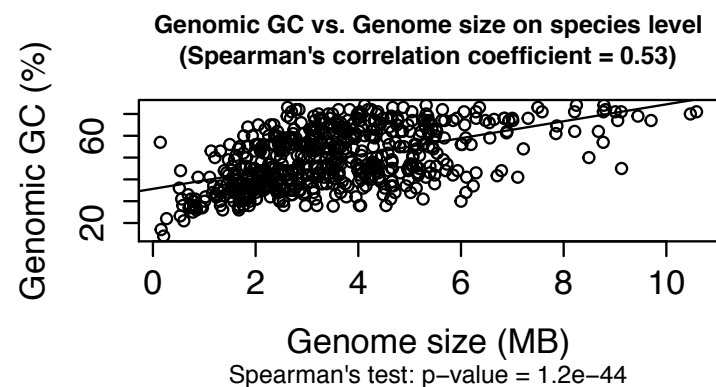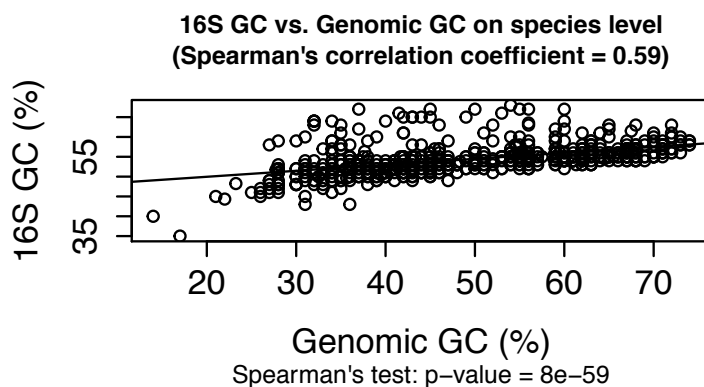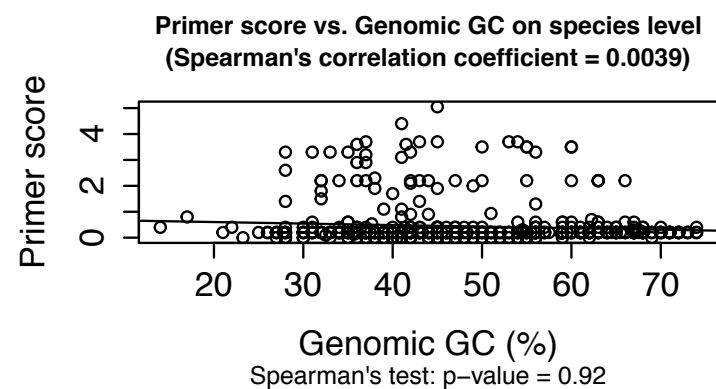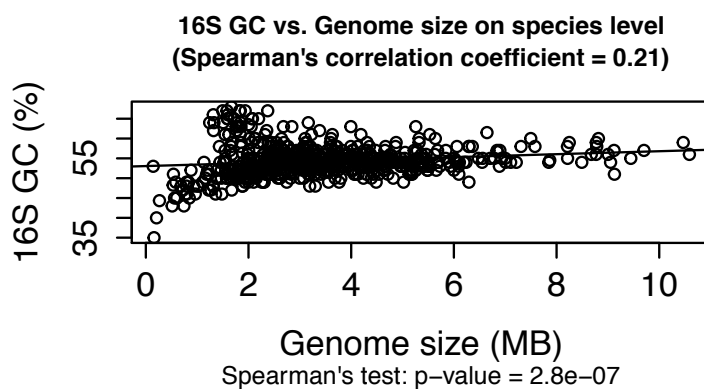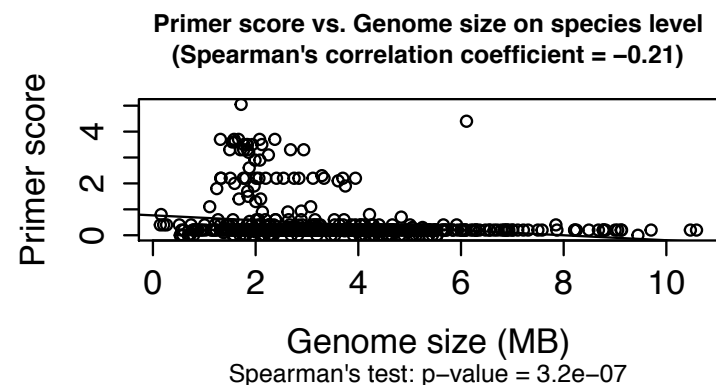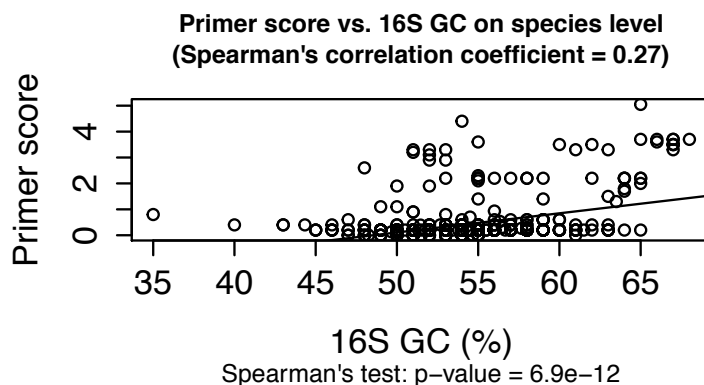

Supplement: S3 Fig — (PDF) [file pone.0124158.s003.pdf]
