# Supplementary material for: New Perspectives on Microbial Community Distortion after Whole-Genome Amplification
Source: PLoS One. 2015 May 26;10(5):e0124158. doi: 10.1371/journal.pone.0124158 (PMC4444113; doi:10.1371/journal.pone.0124158)
Supplement: S2 Fig — (PDF) [file pone.0124158.s002.pdf]

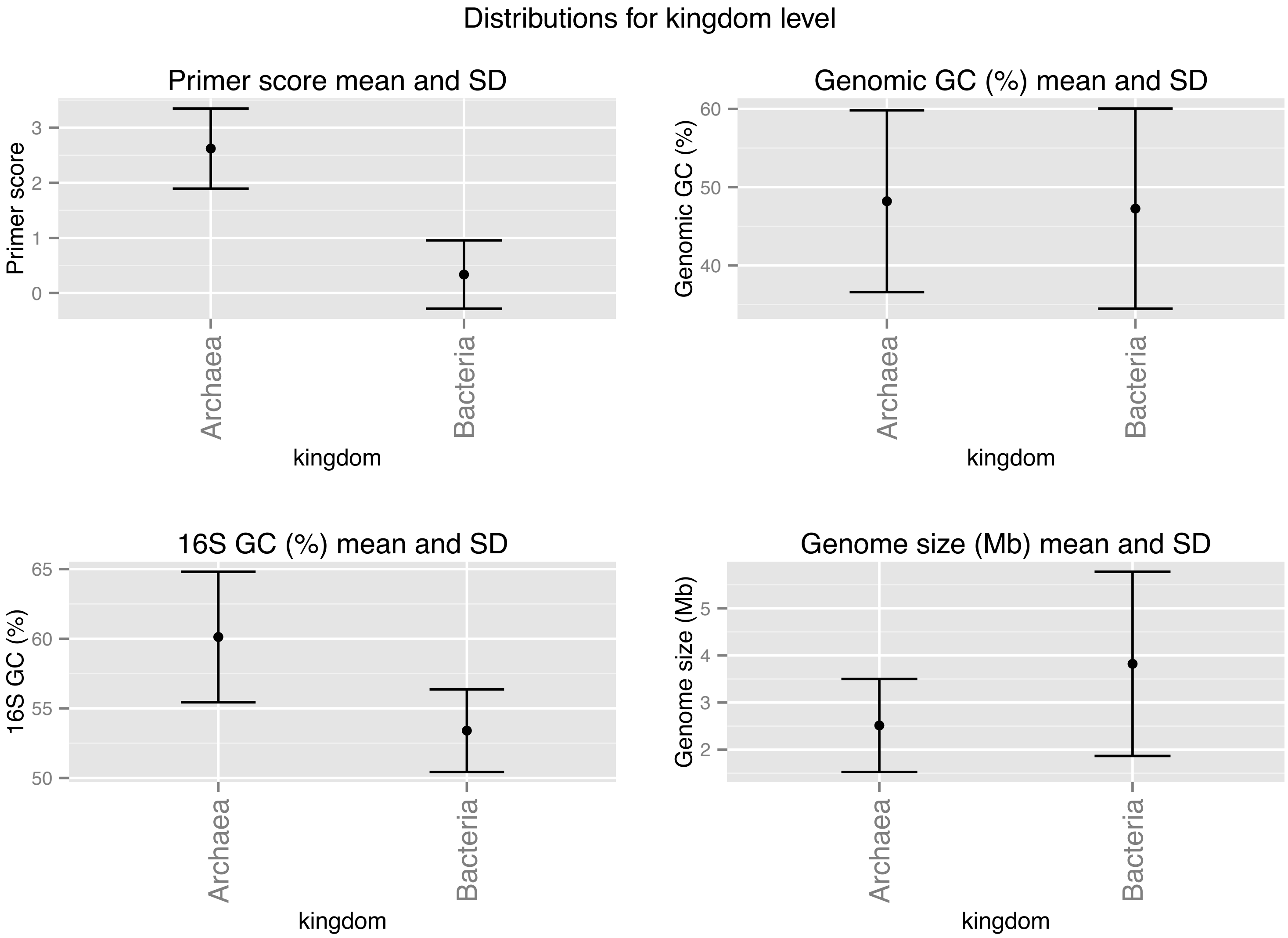

Distributions for kingdom level
Primer score mean and SD
Primer score
3
2
1
0
Archaea
Bacteria
kingdom
Genomic GC (%) mean and SD
Genomic GC (%)
60
50
40
Archaea
Bacteria
kingdom
16S GC (%) mean and SD
16S GC (%)
65
60
55
50
Archaea
Bacteria
kingdom
Genome size (Mb) mean and SD
Genome size (Mb)
5
4
3
2
Archaea
Bacteria
kingdom

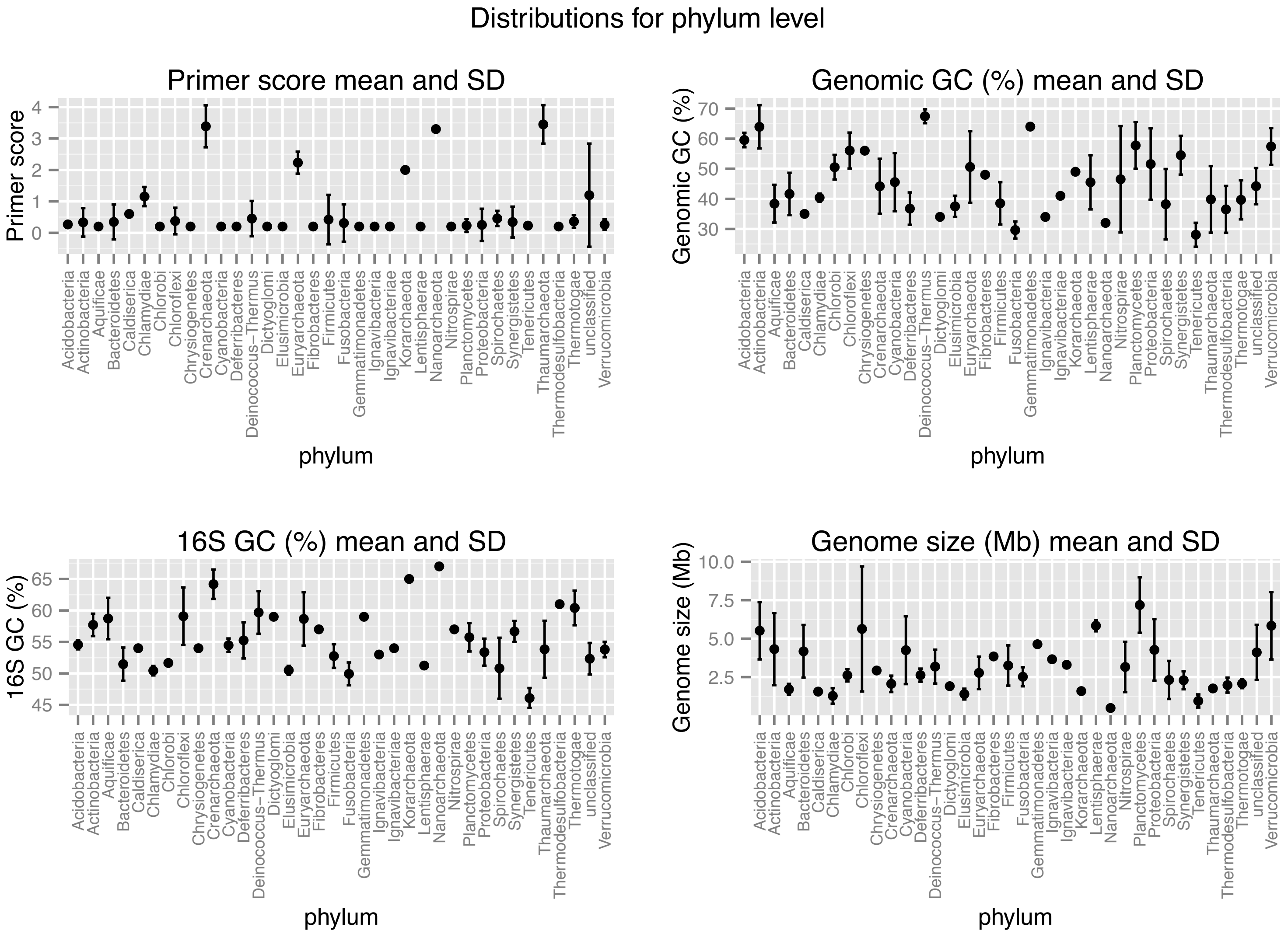
Distributions for phylum level
Primer score mean and SD
Primer score
0 1 2 3 4
Genomic GC (%) mean and SD
Genomic GC (%)
30 40 50 60 70
16S GC (%) mean and SD
16S GC (%)
45 50 55 60 65
Genome size (Mb) mean and SD
Genome size (Mb)
2.5 5.0 7.5 10.0
phylum
Acidobacteria
Actinobacteria
Aquificae
Bacteroidetes
Caldiserica
Chlamydiae
Chlorobi
Chloroflexi
Chrysiogenetes
Crenarchaeota
Cyanobacteria
Deferribacteres
Deinococcus−Thermus
Dictyoglomi
Elusimicrobia
Euryarchaeota
Fibrobacteres
Firmicutes
Fusobacteria
Gemmatimonadetes
Ignavibacteria
Ignavibacteriae
Korarchaeota
Lentisphaerae
Nanoarchaeota
Nitrospirae
Planctomycetes
Proteobacteria
Spirochaetes
Synergistetes
Tenericutes
Thaumarchaeota
Thermodesulfobacteria
Thermotogae
unclassified
Verrucomicrobia

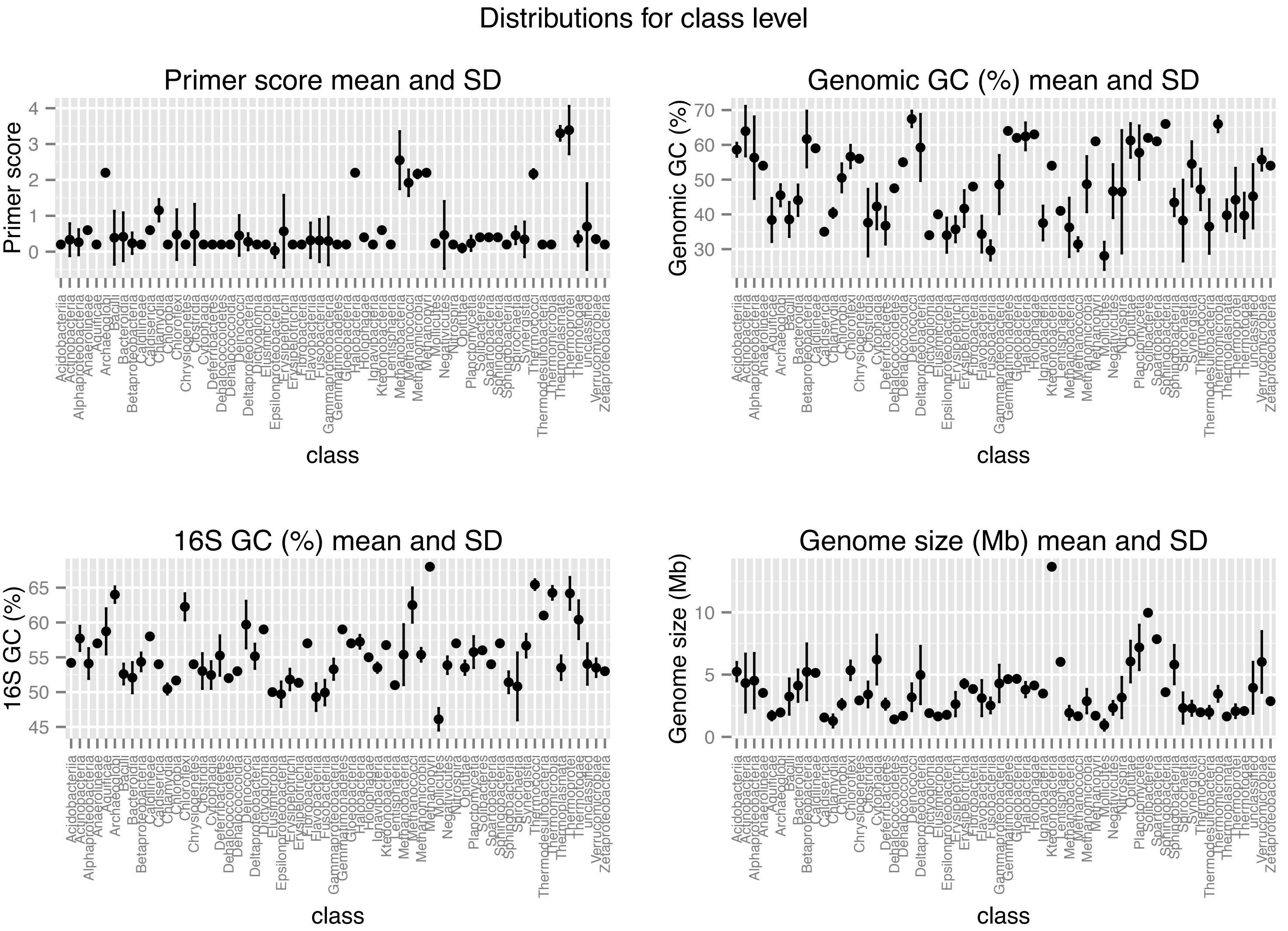
Distributions for class level
Primer score mean and SD
Primer score
class
Genomic GC (%) mean and SD
Genomic GC (%)
class
16S GC (%) mean and SD
16S GC (%)
class
Genome size (Mb) mean and SD
Genome size (Mb)
class

Distributions for order level

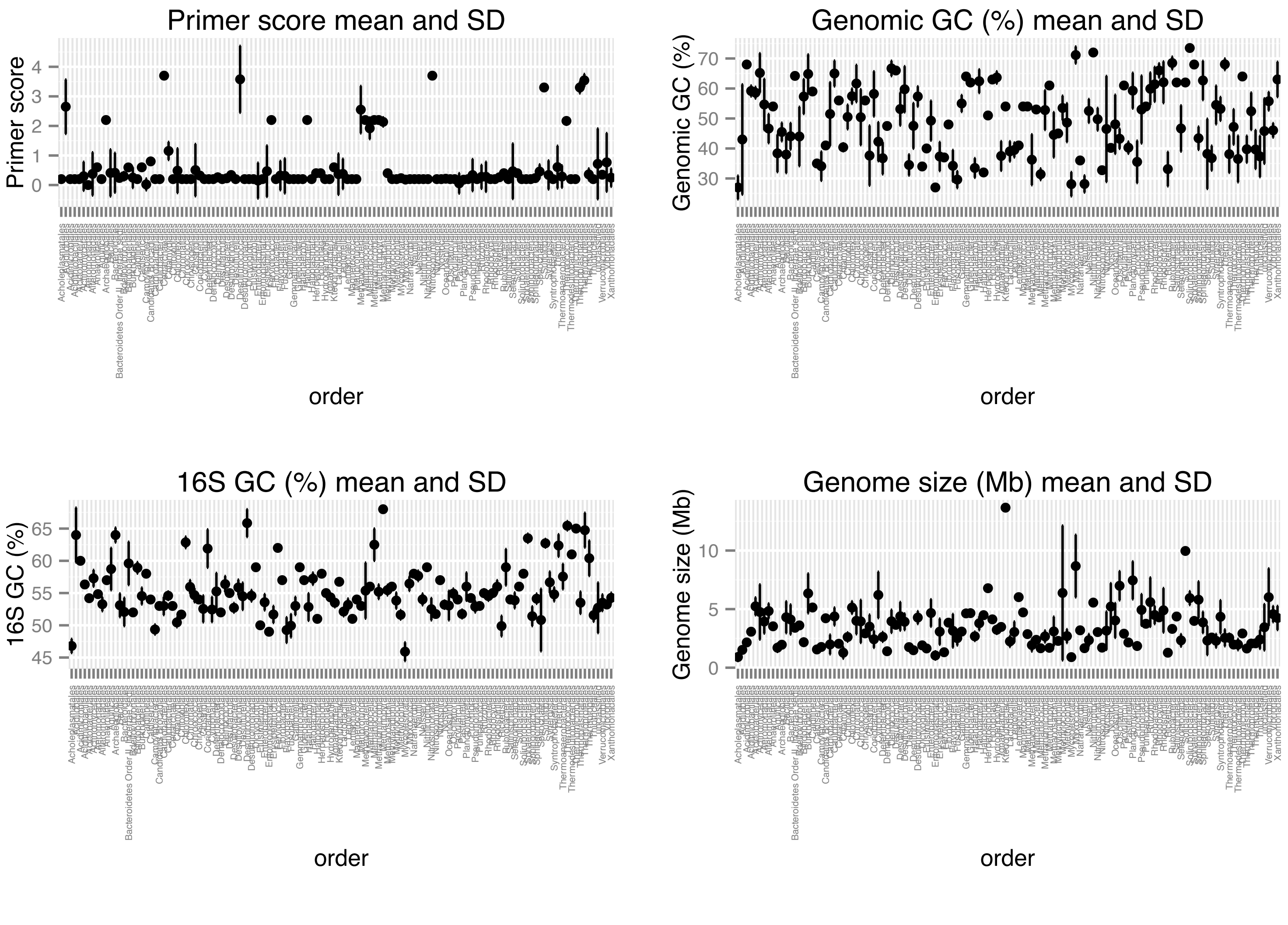

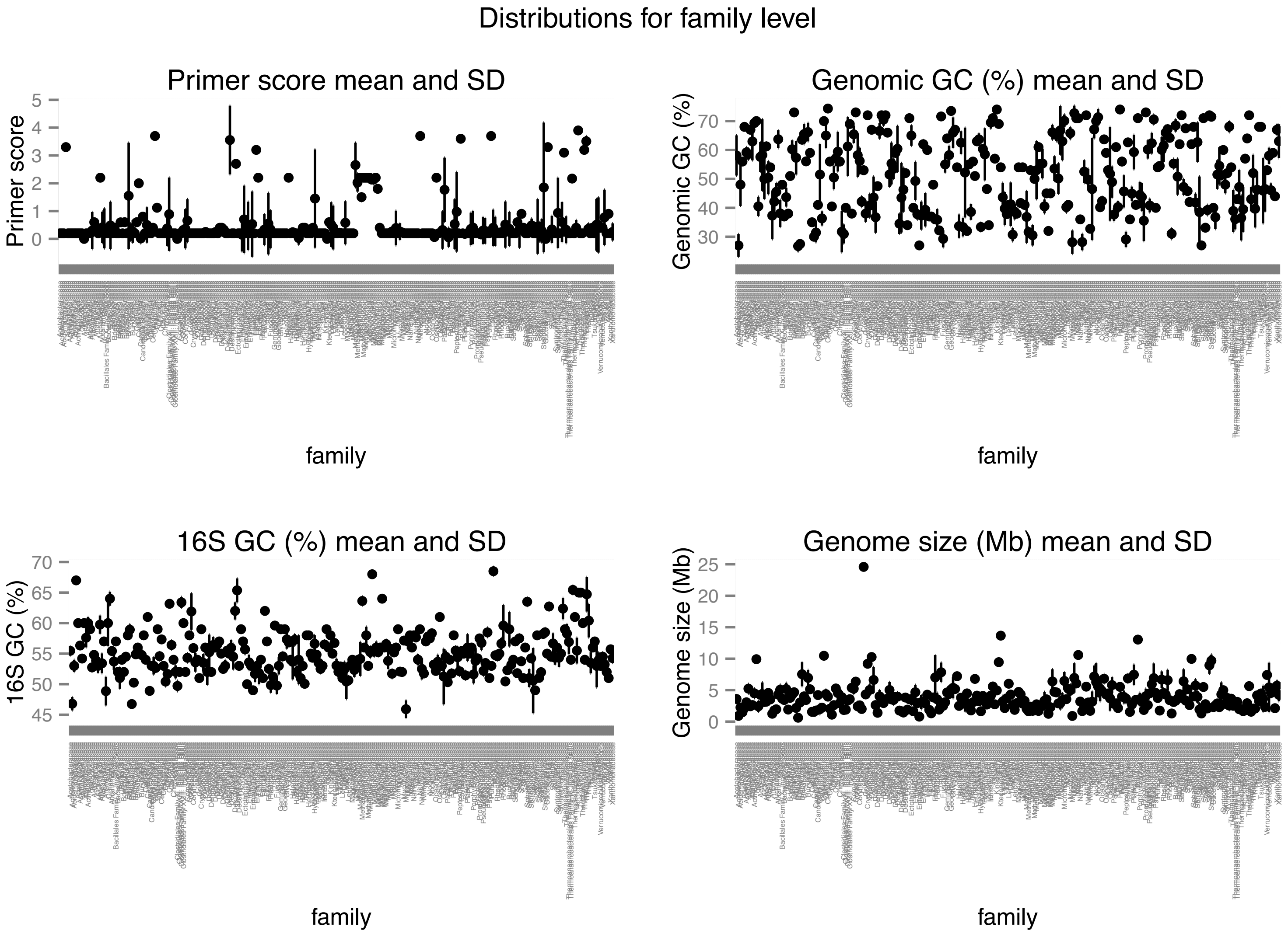
Distributions for family level
Primer score mean and SD
Primer score
0
1
2
3
4
5
family
Genomic GC (%) mean and SD
Genomic GC (%)
30
40
50
60
70
family
16S GC (%) mean and SD
16S GC (%)
45
50
55
60
65
70
family
Genome size (Mb) mean and SD
Genome size (Mb)
0
5
10
15
20
25
family

Distributions for genus level

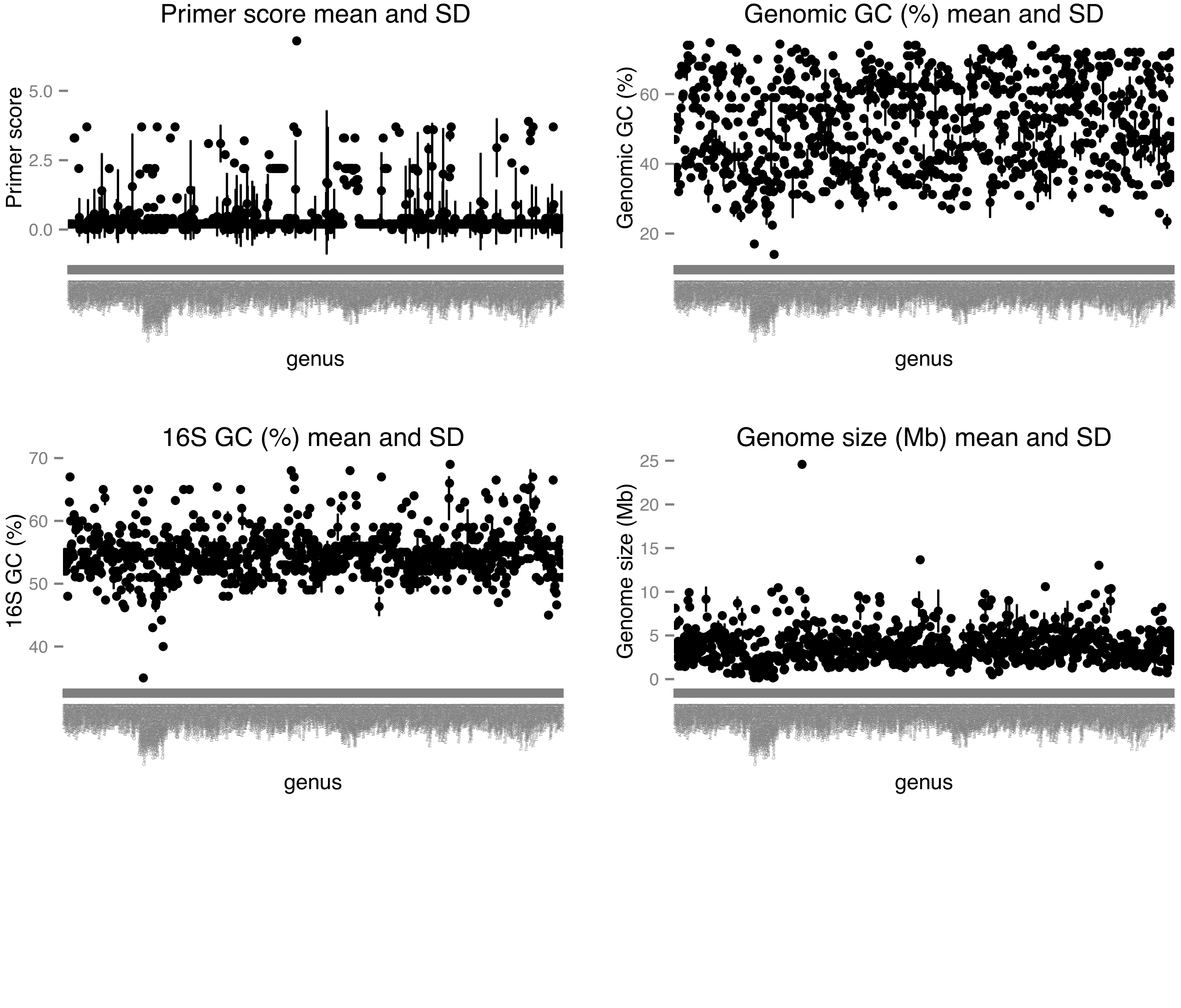

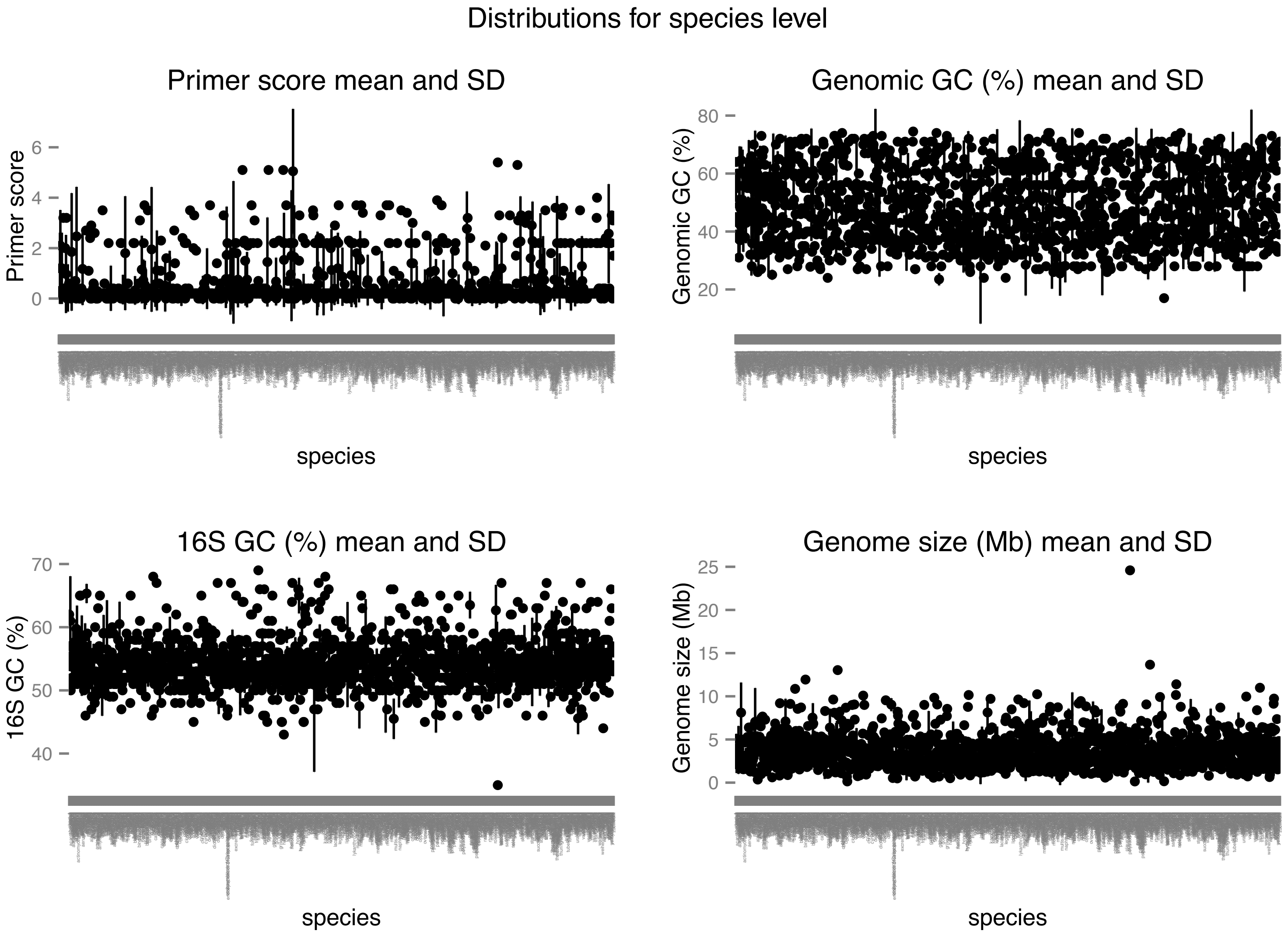
Distributions for species level
Primer score mean and SD
Primer score
0
2
4
6
species
Genomic GC (%) mean and SD
Genomic GC (%)
20
40
60
80
species
16S GC (%) mean and SD
16S GC (%)
40
50
60
70
species
Genome size (Mb) mean and SD
Genome size (Mb)
0
5
10
15
20
25
species
